# Virion content unpacked by long-read sequencing: stress-induced changes in transmitted staphylococcal mobilome due to phage-satellite interactions

**DOI:** 10.1093/nar/gkaf1165

**Published:** 2025-11-08

**Authors:** Tibor Botka, Soňa Smetanová, Adam Vinco, Eliška Kučerová, Kristína Rovňáková, Alena Siváková, Ivana Mašlaňová, Roman Pantůček

**Affiliations:** Department of Experimental Biology, Faculty of Science, Masaryk University, Brno 625 00, Czech Republic; Department of Experimental Biology, Faculty of Science, Masaryk University, Brno 625 00, Czech Republic; RECETOX, Faculty of Science, Masaryk University, Brno 625 00, Czech Republic; Department of Experimental Biology, Faculty of Science, Masaryk University, Brno 625 00, Czech Republic; Department of Experimental Biology, Faculty of Science, Masaryk University, Brno 625 00, Czech Republic; Department of Experimental Biology, Faculty of Science, Masaryk University, Brno 625 00, Czech Republic; Department of Microbiology, St Anne’s University Hospital and Faculty of Medicine, Masaryk University, Brno 625 00, Czech Republic; Department of Experimental Biology, Faculty of Science, Masaryk University, Brno 625 00, Czech Republic; Department of Experimental Biology, Faculty of Science, Masaryk University, Brno 625 00, Czech Republic

## Abstract

The evolution of the virulence and antibiotic resistance of staphylococci, important opportunistic pathogens, is strongly determined by their mobilome, which can spread by phage virions or small-headed particles resulting from the hijacking of helper phage machinery by phage satellites named phage-inducible chromosomal islands (PICIs). Despite known mechanisms of the formation of transducing particles, it has not yet been possible to analyze their DNA content at the single-virion level. Using the *Staphylococcus epidermidis* model and long-read nanopore sequencing, we determined the sequence structure and ratio of phage and PICI genophores, plasmid, and bacterial DNA packaged in normal and small-headed virions. It was shown that the ratios vary mainly depending on the helper phage and the antimicrobial used for induction. When the effect of a strictly lytic phage and its combination with ciprofloxacin on a packaged mobilome was analyzed, no significant increase in mobilome dissemination was observed compared to antibiotics alone. Here, we demonstrate a novel approach for the analysis of transduced bacterial mobilome and show *in vitro* that lytic phage-based therapeutic strategies do not increase the risk of mobile genetic element transfer.

## Graphical Abstract

**Graphical Abstract:**
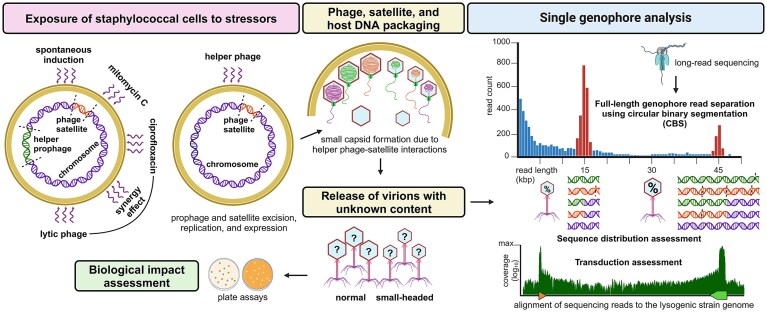

## Introduction

Staphylococcal infections pose a serious problem in hospitalized patients, especially when complicated by antibiotic resistance [1, 2]. While *Staphylococcus aureus* is a known coagulase-positive opportunistic pathogen, coagulase-negative *Staphylococcus epidermidis* has been assumed to be a common human commensal, part of normal skin microbiota [3]. However, even *S. epidermidis* has recently become an increasing contributor to nosocomial infections [4] that can be complicated by increasing invasivity [5] and biofilm formation [2]. The evolution of staphylococci is significantly driven by the acquisition of mobile genetic elements (MGEs), a source of pathogenicity-associated genes that can spread by intra- and interspecies horizontal gene transfer, leading to the emergence and evolution of drug-resistant strains [6, 7].

Temperate phages often carry accessory genes that increase the virulence of lysogenic hosts [8–11] and can mediate generalized or specialized transduction [12–14]. In *S. aureus* prophages, lateral transduction was discovered [15] when the packaging was initiated from the prophage *pac* site before the prophage excision. The packaging via a headful mechanism then continues by encapsidating the adjacent bacterial DNA up to hundreds of kilobases downstream from the prophage sequence [16].

Prophage excision can be triggered by SOS response-inducing antimicrobials or phage infection [17, 18], causing the subsequent spread of phage genome and accessory genes in the environment by lysogenic conversion [19] or by the acquisition of transduced MGE [12, 20–24]. This process can lead to the emergence of bacterial strains that are resistant to the applied therapy. Therefore, in the context of mobilome induction, the safety of all therapeutic approaches, including phage therapy (which is experiencing increased interest), should be considered.

Besides common MGEs such as plasmids and prophages, a family of phage-inducible chromosomal islands (PICIs), which frequently harbor genes for virulence and resistance factors [25–27], can be mobilized by helper phages [28]. Their mobilization can be associated with helper phage capsid remodeling, which results in the formation of small-headed virions [13, 29, 30]. This causes a reduction in phage spread in the host population due to a decreased number of virions carrying the phage genome and the fact that small-headed particles are unable to carry the entire phage genome, so infection by such a virion does not lead to productive phage infection [31, 32].

Since PICIs employ the same mechanism of DNA packaging as helper phages, they can mediate generalized [33] and lateral transduction [34]. As the phage terminase small subunit (TerS) and the PICI TerS recognize different *pac* sequences, PICIs expand the transduction repertoire of phages.

Previous studies have assessed transduction by quantifying the frequency of successfully transduced elements carrying selection markers, particularly antibiotic resistance genes, using the plaque-forming units titer [13, 24, 35], which does not reflect particles carrying non-phage DNA and biases the estimation of the transduction dynamics. Moreover, such traditional approaches are inherently limited as they overlook the broader spectrum of MGEs that can be transduced and focus only on the final step of transduction, which may be influenced by host anti-MGE mechanisms, both inhibitory [36, 37] and promotive [38]. Consequently, these methods used alone fail to provide a comprehensive view of the mobilome dynamics and the true extent of horizontal gene transfer.

Here we introduce a novel, sequencing-based approach that overcomes these limitations by directly identifying and quantifying full-length genophores packaged in individual virions, including both normal and small-headed particles modified by PICIs. Using a single-genophore resolution, we analyze the packaging mechanisms and distribution of phage, PICI, plasmid, and chromosomal DNA under different stress factors. Notably, the study demonstrates that *in vitro* treatment combining lytic phage and antibiotics does not increase the risk of MGE spread compared to antibiotics alone, providing important insights into the safety of phage-based therapy.

## Materials and methods

### Biological resources

Two helper *S. epidermidis* siphophages were used: phage 48 [39] and phage E72m5 (in this study). Phage 48 was propagated on *S. epidermidis* strain SE48 [39], and phage E72m5 on *S. epidermidis* strain 1457 [40]. Phage 812h1 [41] of the *Kayvirus* genus was used to assess the effect of phage-based therapy on transduction compared to antibiotic treatment. All bacterial strains used in this study are listed in Table 1.

**Table 1.** Properties and use of *S. epidermidis* and other staphylococcal strains in this study.

	MGE content				
Strain	SeCI_SE48_	Prophage	Plasmid	Properties and use	Reference
SE48	YES	NO	pSE48_1, pSE48_2	Propagation strain for phage 48; infection system with phage 48	Dean *et al.* [39]
SE48L	YES	48	pSE48_1, pSE48_2	Lysogen of strain SE48; used in induction systems	This study
1457	NO	1457	p1457	Propagation strain for phage E72m5; sensitive to phage 812h1	Galac *et al.* [40]
1457(SeCI_SE48_+)	YES	1457	p1457	Infection system with phage E72m5	Fišarová *et al.* [13]
1457LA	YES	E72m5	p1457	Used in induction systems	This study
1457LA_ermC	YES^a^	E72m5	p1457	Used for transduction experiments with erythromycin selection	This study
1457c	NO	NO	p1457	Cured for prophage 1457; control infection system with phage E72m5	This study
*S. aureus* 1137	NO	NO	NO	Propagation strain for phage 812h1; resistant to phage E72m5	AUMED s.r.o., Czechia
*S. cohnii* A6C	NO	NO	2.7-kb	Sensitive to phage E72m5; resistant to phage 812h1	Dean *et al.* [39]

a SeCI_SE48_ with the incorporated *ermC* gene. GenBank acc. nos. of listed strains: SE48 (CP066303), SE48L (CP180175), 1457 (CP020463), 1457(SeCI_SE48_+) (CP180176), 1457LA (CP180177), 1457LA_ermC (CP194848), and 1457c (CP180178). GenBank acc. nos. of listed MGEs: SeCI_SE48_ (MW368309); prophages 48 (MW364972), 1457 (CP020463), and E72m5 (PV036961); plasmids pSE48_1 (CP066304), pSE48_2 (CP066305), and p1457 (CP020462).

Lysogenization of *S. epidermidis* strains was performed as described previously [8]. Genome editing was performed using the plasmid pIMAY, as described previously [42]. The sequences for homologous recombination were amplified using the primers listed in Supplementary Table 1. For the deletion of prophage 1457 from strain 1457 (pos. 1 793 020–1 835 461 bp in GenBank reference genome CP020463.1), pIMAY and the insert were cleaved using *SalI*-HF and *XmaI* (New England Biolabs, USA) and ligated using the Quick Ligation Kit (New England Biolabs), obtaining strain 1457c.

Strain 1457LA_ermC was prepared by replacement of a part of the SeCI_SE48_ sequence in position 889 868–891 129 bp of the strain 1457LA genome (CP180177.1) by an *ermC* cassette from pCN51 vector (KR781468, pos. 2101–3361 bp) [43] of the same size. The *ermC* gene and the homologous arms were assembled with the linearized vector using NEBuilder HiFi DNA Assembly Master Mix (New England Biolabs). In both cases, the obtained constructs were transformed into *Escherichia coli* DC10B and verified using the Sanger sequencing service (Eurofins Genomics, Germany). The corresponding construct was then electroporated into strain 1457 for deletion of prophage 1457 and 1457LA for size-conserved insertion of the *ermC* gene.

### Preparation of phage lysates, phage enumeration, and efficiency of plating determination

The high-titer phage lysates were prepared using phages and their propagation strains in meat-peptone broth (MPB) prepared from 13 g of nutrient broth (Oxoid, UK), 3 g yeast extract (Oxoid), 5 g peptone (Oxoid), and distilled water to a final volume of 1 l (pH 7.4), and supplemented with CaCl_2_ to a final concentration of 2 mM. The input ratio (IR or MOI_INPUT_) of phages was 0.1. Bacterial debris was removed by centrifugation at 3100 × *g* for 30 min, and supernatants were filtered using a polyethersulfone membrane filter with a pore size of 0.45 μm (TPP, Switzerland).

For phage titer determination by the double-layer agar (DLA) method, respective dilutions of phage lysates were mixed with overnight bacterial cultures and soft 0.7% meat-pepton agar (MPA) with the addition of CaCl_2_ to a final concentration of 2 mM. MPA (1.5%) plates were overlayed with this suspension and incubated at 37°C overnight. The number of plaque-forming units per milliliter (PFU/ml) was assessed as the mean of three technical replicates for each dilution. The efficiency of plating was calculated as the ratio of titers obtained on the strains of interest.

### Transmission electron microscopy

For electron microscopy, virions were purified in a CsCl density gradient [44] and then diluted to an A_280_ of 0.5–1.0. Negatively stained samples were prepared by applying 4 μl of the diluted sample onto copper grids coated with a 12-nm carbon layer and stained with 2% uranyl acetate. Samples were observed using a Tecnai F20 electron microscope (Thermo Fisher Scientific, USA) operated at 200 kV with ×50 000 magnification.

### Minimum inhibitory concentration of antimicrobials and induction efficiency determination

Antimicrobial susceptibility testing was performed using the broth microdilution method (Trios, Czechia) in accordance with ISO standard 20776–1:2019. The minimum inhibitory concentration (MIC) of gentamicin, oxacillin, and ciprofloxacin was interpreted in accordance with EUCAST guidelines (version 14.0, 2024) [45]. Methicillin-resistant *S. aureus* ATCC 43300 was used as a control strain. MIC of mitomycin C (SERVA Electrophoresis GmbH, Germany) was assessed as a concentration that fully inhibited bacterial growth after 18 h of incubation at 37°C. The induction of prophages by these antimicrobials was evaluated using a drop plaque assay with overnight incubation at 37°C of the lysates obtained using 1.0×, 0.75×, and 0.5× MIC of the respective antibiotics and 2 mg/l of the final concentration of mitomycin C (4× MIC for strain SE48L and 8× MIC for 1457LA). The number of PFU/ml was assessed by the DLA method as the mean of three technical replicates.

### Adsorption assay

An overnight culture of the strain 1457LA was inoculated into fresh MPB and cultivated with aeration at 37°C to an OD_600_ = 0.6 (≈ 10^8^ CFU/ml). The culture was mixed with 812h1 phage lysate (IR = 10) and CaCl_2_ to a final concentration of 2 mM and cultivated at 37°C with aeration. Samples were taken at 0, 2, 5, 10, 15, and 20 min after inoculation. For adsorption assay of phage E72m5 on strains 1457 and A6C (IR = 0.5), brain heart infusion (BHI) broth (Oxoid), OD_600 _= 1.9–2.0 (≈ 1 × 10^9^ CFU/ml), CaCl_2_ final concentration of 4 mM, and sampling at 0, 2, 5, 10, 20, and 30 min were used to follow the same conditions as used in the transduction assays. The percentage of free virions in the supernatant after cell pelleting at 9700 × *g* for 2 min was determined using the DLA method in three technical replicates.

### Preparation of induced phage lysates

Induced lysates were prepared using 500 ml of fresh bacterial cultures in MPB grown to OD_600 _= 0.25 and 2 mg/l of the final concentration of mitomycin C or 1× MIC of ciprofloxacin. After incubation at 37°C for 2 h with gentle aeration (80 rpm), the cultures were stored in the dark, statically, at room temperature overnight. Spontaneously induced lysates were prepared from 1 l of bacterial culture without the addition of an inducing agent. Bacterial debris was removed by centrifugation at 3100 × *g* for 30 min, and supernatants were filtered using a polyethersulfone membrane filter with a pore size of 0.22 μm (TPP, Switzerland).

### Treatment of strain 1457LA with a lytic *Kayvirus* phage

A shaken overnight culture of strain 1457LA was inoculated into 100 ml of fresh MPB and cultured to OD_600_ ≈ 0.25. Phage 812h1 (IR = 10 or 100) or phage 812h1 and 1× MIC of ciprofloxacin were added. This was followed by incubation with gentle shaking (80 rpm) at 37°C for 2 h. Lysis was finalized in the dark, statically, at room temperature overnight. Bacterial debris was filtered out, and titers of induced phage E72m5 and lytic phage 812h1 and viable cell counts were determined for six biological replicates, which were measured in technical triplicates for each dilution. To distinguish the growth characteristics of the therapeutic phage 812h1 and induced phage E72m5, *S. aureus* strain 1137 (sensitive to phage 812h1 and resistant to phage E72m5), *Staphylococcus cohnii* strain A6C (sensitive to phage E72m5 and resistant to phage 812h1), and *S. epidermidis* strain 1457 (sensitive to both phages) were used. To determine viable cell counts, tryptone soya agar (TSA; Oxoid) was used.

### Transductions

Transducing lysates from spontaneous and ciprofloxacin (1× MIC) induction, phage 812h1 infection (IR = 10 or 100), and the combined treatment with phage 812h1 and ciprofloxacin were prepared as described earlier using *S. epidermidis* strain 1457LA_ermC. An overnight culture of recipient strain *S. cohnii* A6C grown in BHI broth was diluted to OD_600 _= 1.9–2.0 (≈ 1 × 10^9^ CFU/ml) and enriched with CaCl_2_ to a final concentration of 4 mM. Tenfold dilutions of transducing lysates were mixed with an equal volume of recipient and incubated at 37°C for 30 min. Aliquots of each transduction assay were plated in technical triplicate on BHI agar plates supplemented with sodium citrate at a final concentration of 20 mM and erythromycin at a final concentration of 5 μg/ml and incubated at 37°C for 36 h. For each treatment, the titer of phage E72m5, the number of transductant-forming units per milliliter of transducing lysate (TFU/ml), and the transduction frequency (TFU/PFU) were calculated for each of the six biological replicates, along with the descriptive statistics.

### Extraction of DNA from virions

Lysates were concentrated using Amicon 30 kDa MWCO (Sartorius) to a final volume of 40 ml and centrifuged at 64 000 × *g* for 2.5 h at 6°C using a Beckman Avanti J-30I high-speed centrifuge. Each pellet was spontaneously resuspended in 400 µl of DNase buffer (2.5 mM MgCl_2_, 0.1 mM CaCl_2_, 10 mM Tris–HCl, pH 7.5) overnight at 4°C. The samples were treated with 2 µl DNase I (2500 U/ml; Thermo Scientific, cat. no.: 90083) and 5 µl RNase A (10 mg/ml; Thermo Scientific) for 90 min at 37°C. Next, 10 µl Proteinase K (20 mg/ml; Sigma–Aldrich) and 30 µl of 10% sodium dodecyl sulfate (SDS) were added, followed by incubation on a shaking thermoblock at 55°C, 250 rpm for 15 min. The DNA was extracted using a Genomic DNA Clean & Concentrator-10 kit (Zymo Research) according to the manufacturer’s instructions. The absence of external DNA was confirmed by the polymerase chain reaction (PCR) detection (Supplementary Fig. 1) of control DNA of *Pseudomonas* phage LUZ19 [46], added in a total amount of 30 ng to the samples before treatment. Primers LUZ19-mcp_F (CTTCGACATGCGCAAGGAAG) and LUZ19-mcp_R (CGGTCAGGTCCAGTTTCTCC), targeting the gene encoding the major capsid protein of phage LUZ19, were used for external DNA detection. Primers endlys_F (CCATAGAACGGTGAGGACAGG) and endlys_R (TGGAGTGGGTGAGAATCCCTT), targeting endolysin gene of phage 812h1 [41]; and primers SeCI_F (CGTTGAGGGCTTGAAATGGG) and SeCI_R (GCACCTAACACTTGGCGTTT), targeting *gp12* and *gp13* of SeCI_SE48_ [13], were used for internal control (Supplementary Fig. 1). The sequencing data obtained were also mapped on the phage LUZ19 genome sequence (NC_010326) using Minimap2 v2.24 plug-in in Geneious Prime v2025.2.2 (https://www.geneious.com/) with the following setup: Data Type: Oxford Nanopore (more sensitive), K-Mer Length: 28.

### Bacterial DNA extraction

The bacterial DNA was isolated as previously described [13] with some modifications. First, 10 ml of stationary overnight bacterial culture cultivated in TSB at 37°C was centrifuged at 3000 × *g* and 10°C for 10 min, washed with 5 ml of wash solution [10 mM Tris–HCl, 10 mM ethylenediaminetetraacetic acid (EDTA), 10 mM EGTA, 1 M NaCl, pH 7.5], and resuspended in Tris-EDTA buffer with lysostaphin (50 μg/ml; Sigma–Aldrich), achromopeptidase (1000 U/ml; Sigma–Aldrich), mutanolysin (40 U/ml; Sigma–Aldrich), and RNase A (200 μg/ml; Thermo Scientific) in a total volume of 500 μl, followed by incubation for 1–2 h at 37°C until lysis appeared. Then, 100 μl of 10% SDS and 20 μl of proteinase K (20 mg/ml; Sigma–Aldrich) were added, and the sample was incubated for 30 min at 55°C, followed by cooling to 4°C and centrifugation at 9700 × *g* for 10 min. The DNA was extracted from the supernatant using a Genomic DNA Clean & Concentrator-25 kit (ZymoResearch) according to the manufacturer’s instructions.

### Whole-genome sequencing, annotation, and mapping

Libraries of DNA from phage particles were prepared using a Ligation Sequencing Kit V14 (SQK-LSK114; Oxford Nanopore Technologies, UK) according to the manufacturer’s instructions. Libraries were sequenced with FLO-FLG114 flow cells (R10.4.1) in a MinION device (Oxford Nanopore Technologies, UK). The device was controlled with the software MinKNOW v23.11.7 (Oxford Nanopore Technologies, UK), which was also used for base-calling (super accuracy mode) and trimming. Biological triplicates were prepared for all evaluated systems. The obtained sequences of packaged DNA were mapped to reference sequences of the source MGEs and bacterial chromosomes. Sequencing read mapping and statistics were computed using Geneious Prime v2025.2.2 (https://www.geneious.com/) and the Geneious mapper plug-in (Minimum quality mapping: 10). The specific mapping parameters and reference genomes used are listed in the corresponding supplementary tables.

To obtain whole bacterial and phage genomes, the Oxford Nanopore Technologies (ONT) platform, using a Rapid Barcoding Kit V14 (SQK-RAD114) or a Ligation Sequencing Kit V14 (SQK-LSK114), and the Illumina platform (Illumina, USA) were used. Illumina sequencing was performed with a NextSeq sequencer using a NextSeq 500/550 Hi Output Kit v2.5 (75 cycles) and a 150-bp paired-end mode. Reads were trimmed using the sliding window model in Trimmomatic Galaxy v0.38.1 [47]. Complete genomes were obtained via hybrid assembly using Unicycler Galaxy v0.5.0 [48] or Flye v2.9.5 [49] for ONT-only sequenced genomes. Phage E72m5 genome was annotated using Pharokka v1.3.2 [50] with manual inspection using BLAST (http://blast.ncbi.nlm.nih.gov/). Bacterial genomes were annotated using the NCBI Prokaryotic Genome Annotation Pipeline [51].

### Determination of normal and small-sized genophore ratio

DNA samples were analyzed using an Advanced Analytical Fragment Analyzer (Agilent) 12-capillary instrument using an Agilent DNF-468 HS Genomic DNA 50 kb Kit (DNF-468-0500). Samples were first diluted to a concentration of 1–10 ng/µl and then mixed with the diluent marker according to the user manual. Peak delineation was evaluated using the instrument’s proprietary software PROsize with manual correction.

### Identification of full-length genophores and determination of the sequencing read fraction with an ambiguous origin, and other statistical analyses

**Figure 1.**
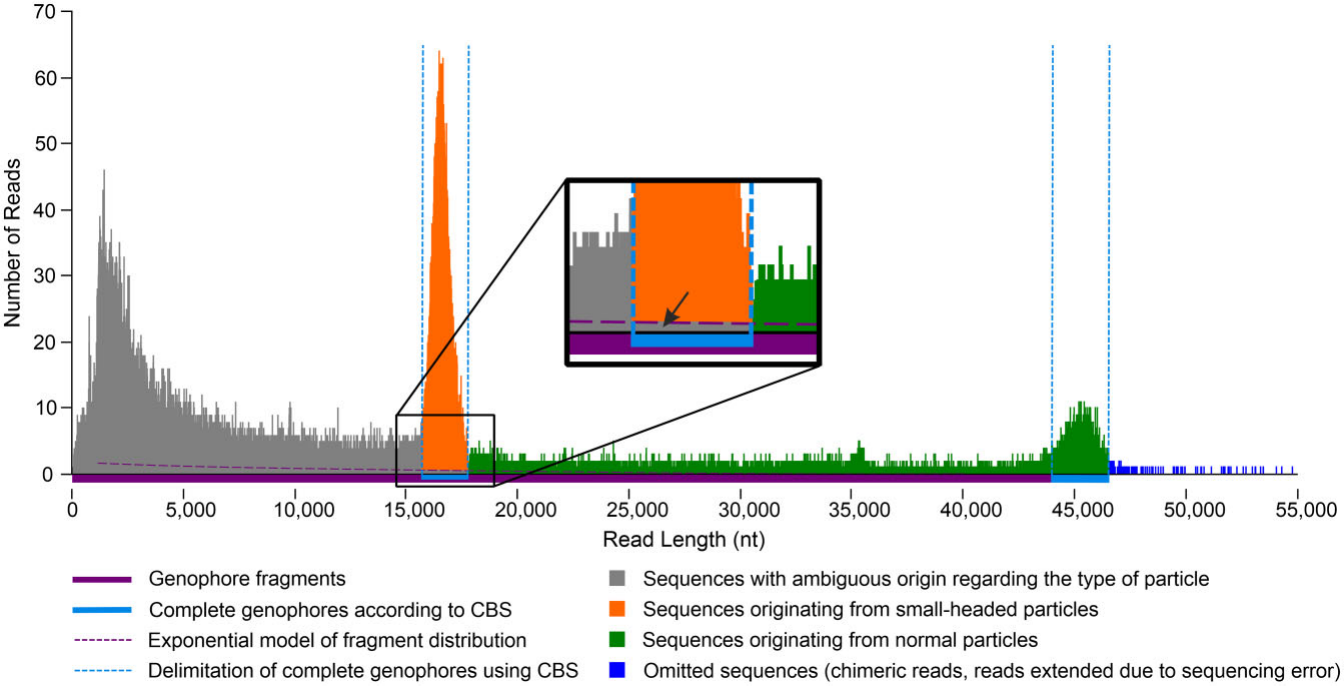
Distribution of sequenced reads and determination of complete genophores from small and normal capsids using CBS. The fraction of sequences with ambiguous origin in the peak of the genophores carried in small capsids (indicated by a black arrow) was estimated using an exponential function. Most sequencing lengths have very low abundance (0 or 1); they are densely clustered on the *x*-axis, which causes the exponential model to appear low and makes the abundant sequencing lengths seem visually overrepresented. The figure displays data from the phage E72m5 infection system (NCBI SRA acc. no. SRR32323969).

Circular binary segmentation (CBS) [52, 53] was used to distinguish reads representing full-length genophores in normal and small-headed virions from a background consisting of fragmented DNA (Fig. 1). The robustness of the CBS method setting was tested under different parameters. The results are accessible on Zenodo (zenodo.org). Based on the literature search [52, 53] and our robustness testing results, the significance level for the test to accept change points (*alpha*) was finally set to 0.01, the minimum number of markers for a changed segment (*min_width*) was 2, and the number of permutations (*nperm*) was 10 000. Using CBS, sequencing data were divided into homogeneous segments with significantly different average read counts to define genophores from normal and small capsids statistically. The range of genophore sequence lengths was determined so that the segment with the minimum (or maximum) sequence length in the peak formed by the genophores of a given capsid type had an average read count ≥2. If no segment in the peak was ≥2 (which was often the case for genophore sequences in normal capsids), the span of the segment with the highest average read count value was used. Length ranges were described for each biological replicate separately and for the combined data from infection systems. An exponential regression model was used to calculate the relative number of sequences with ambiguous origin in terms of particle type in a set of complete genophores from small-headed particles determined by CBS (Fig. 1). Sequencing data of each biological replicate with lengths ≥1 000 and ≤40 000 nt (excluding those within the range of genophores from small-headed particles) was used to build the model, from which the number of these sequences was predicted.

Differences in median values and Cliff’s Delta [54] were used to characterize differences between genophore length ranges from different biological replicates (statistical testing was not performed because large sample sizes make even negligible differences statistically significant). A *t*-test was used for statistical testing of differences between the relative abundances of genophores of different origins in released normal and small-headed particles, as well as between the relative content of phage and SeCI_SE48_ genome copies in the sequencing data. Differences between counts of released virions of infecting phage 812h1, induced phage E72m5, and viable cell counts of 1457LA culture after treatments were tested using Analysis of variance with the Games–Howell *post-hoc* test [55] due to unequal variances. Values were logarithmically transformed, and outlying values were excluded prior to testing to obtain normally distributed data. Differences in the number of TFUs between various treatments were analyzed using the nonparametric Kruskal–Wallis test followed by Dunn’s post-hoc test, due to the high frequency of zero and low values. For the purposes of statistical testing, values below 10 TFU/ml (≈ LOD; limit of detection) were replaced by the constant 10/√2 [56].

Statistical analyses were performed in R v4.3.2 [57] using additional R packages PSCBS [52, 53] (CBS algorithm), ggplot2 [58] (various graphs), and rstatix [59] (statistical testing).

### Phage-antibiotic interaction assessment

Experimental data on viable cell count (CFU/ml) and prophage induction (PFU/ml) of six biological replicates were converted to percentages, with 100% assigned to the highest observed value in both cases. These data were arranged according to the SynergyFinder v3.12.0 manual [60] and processed in R v4.3.3 [57] with the additional package synergyfinder v3.12.0 [61], also used for descriptive statistics calculation. For data processing, the input data were transformed and processed using a built-in “viability” option. The phage-antibiotic interaction was calculated using Bliss, zero interaction potency (ZIP), highest single agent (HSA), and Loewe additivity models and visualized.

## Results

### Model systems for staphylococcal mobilome analysis

To analyze the distribution and structure of the packaged mobilome after phage infection, we chose two source *S. epidermidis* strains (Table 1) carrying previously characterized PICI SeCI_SE48_ [13], which is a suitable model MGE due to its high transducibility and similarity with various PICIs in genes affecting integration, transcription, or packaging. The first included strain, SE48, is a natural carrier of SeCI_SE48_ and a propagation strain for helper phage 48. The second strain used was 1457 with integrated SeCI_SE48_, designated 1457(SeCI_SE48_+). This strain served as a host for phage E72m5, a spontaneous mutant of phage E72 that is unable to propagate on 1457(SeCI_SE48_+) [13]. To analyze the distribution of the mobilome after prophage induction, we prepared a lysogen of strain SE48, designated SE48L, containing prophage 48; and a lysogen of strain 1457(SeCI_SE48_+) with phage E72m5, designated 1457LA. This lysogenization resulted in the curing of natural prophage 1457 in 1457LA; thus, there was no interference between prophages 1457 and E72m5 during induction. Strains SE48 and 1457(SeCI_SE48_+) were exposed to infection by transducing helper phages 48 and E72m5, respectively, while the corresponding lysogens were exposed to antimicrobial agents mitomycin C and ciprofloxacin, which were selected based on their highest induction potential (Supplementary Table 2). Strain 1457LA was also treated with the lytic therapeutic phage 812h1 of the *Kayvirus* genus [41] alone or in combination with ciprofloxacin. During the lytic cycle of helper phage E72m5, both normal phage virions and small-headed virions resulting from the interaction with SeCI_SE48_-encoded capsid morphogenesis protein were produced (Supplementary Fig. 2), as occurred with phage 48 [13]. To study the effectiveness of SeCI_SE48_ transduction after each treatment, strain 1457LA_ermC containing SeCI_SE48_ with an inserted erythromycin resistance gene was used as a donor, and *S. cohnii* A6C, which is susceptible to the transducing phage E72m5 and resistant to the lytic phage 812h1, as a recipient.

For each host-phage/prophage system, independent biological triplicates were prepared and analyzed. Phage E72m5 infection of strain 1457c, which does not carry prophages or SeCI_SE48_, served as a control for phage genophore length and distribution determination (Supplementary Tables S3 and S4).

### ONT sequencing-based approach for size and origin determination of packaged genophores

After the external genetic material was eliminated using DNase I, the DNA inside the released virions was extracted and sequenced. The successful external DNA elimination was proved by PCR (Supplementary Fig. 1) and mapping of sequencing reads to the genome sequence of phage LUZ19, whose DNA was added as a control prior to sample treatment, with a negative result. We prepared an ONT sequencing library without the DNA fragmentation step to preserve as many of the full-length DNA molecules packaged in the phage particles as possible. We distinguished full-length genophore sequences originating from normal and small-headed particles from fragmented DNA based on their abundance using the CBS algorithm (Fig. 1 and Supplementary Table 3). This approach enabled us to eliminate the bias caused by DNA fragments of unclear origin in terms of particle type, which may also be parts of the same genophore. The origin of genophores from both particle types was subsequently identified by mapping to reference sequences (Supplementary Table 4). For all replicates, we were able to analyze sequences of complete genophores from small capsids determined using CBS. However, due to fragmentation, it was not possible to use CBS to detect complete genophores from normal capsids in some replicates from induction systems (Supplementary Table 3). Still, analysis of reads ranging from 25 to 46 kb ensured that they originated exclusively from normal capsids, while no two or more reads belonged to the same genophore, and excessively long reads (chimeric or caused by sequencing error) were eliminated.

Due to DNA fragmentation, the portion of reads determined as complete genophores from small-headed particles included a small fraction of fragmented DNA from normal particles. Their abundance was estimated using an exponential function (Fig. 1). Because they could not be individually identified, they could not be accurately quantified or removed. As expected, their proportion was generally higher in induced samples due to a higher level of DNA fragmentation (Supplementary Table 3A), but in all assessed cases, this was below 4.7%. This clearly shows that the analysis of the genophores from small-headed particles was not considerably biased.

We compared the median lengths of genophore reads determined by CBS from biological replicates of all systems. Data obtained from the infection systems of both phages had the highest consistency, as the replicates differed negligibly and exhibited the lowest fragmentation (Supplementary Table 3A). Therefore, the length of the genophores was determined based on these data (Supplementary Table 3B).

The approach used enables the accurate separate quantification of genophores of different origins for each capsid type by size, but not a relative quantification of genophores of both capsid types. The reason for this is that the probability of DNA being sequenced on the ONT platform decreases with increasing length, and also due to the higher probability of fragmentation of larger DNA molecules during library preparation. This led, in most cases, to a multiple overrepresentation of sequenced genophores from small-headed virions (Supplementary Table 4). To determine the proportion of normal and small-headed particles, we used an analysis of isolated DNA with an Agilent Fragment Analyzer. As the quantitative accuracy of the kit used is ±30%, it was a rough estimation (Supplementary Table 5).

### Genophore size determination

**Figure 2.**
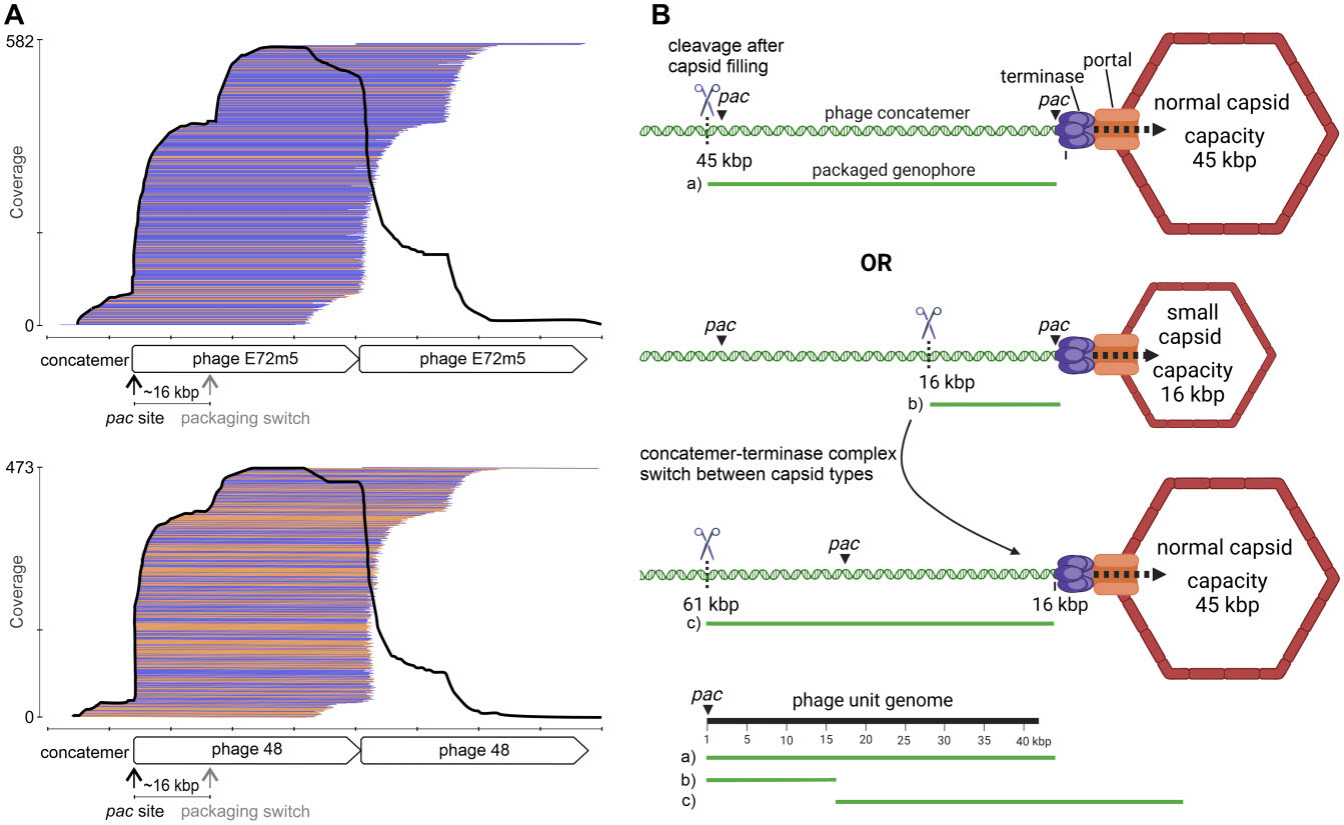
Mapping of phage genophore sequences to the phage concatemer (**A**), and mechanism of the concatemer–terminase complex switching between the two types of capsids (**B**). The obvious bounce in coverage (A) indicates a switch during the packaging of the concatemer, as depicted in part B. Packaging of phage DNA concatemers starts imprecisely from the *pac* site (A and Ba). This results in genophores of ~45 kbp in length with the same or similar start, which can be observed as an increased coverage at the *pac* site (A). However, the concatemers may first be packaged into small capsids. This results in genophores of 16 kbp with a start at the *pac* site (Bb). Subsequently, genophores packaged into normal particles have a similar start at around 16 kbp from the *pac* site (Bc), which is reflected by a typical change in phage genome coverage (A; schematically in part B, below). Analogously, the same mechanism applies to the SeCI concatemers. Data obtained after treatment of strains 1457LA and SE48L with ciprofloxacin were used for mapping (**A**). The color of the mapped genophores indicates their orientation to the reference (blue for forward; orange for reverse). The figure was partly created in BioRender. Botka (2025) https://BioRender.com/l80g597.

**Figure 3.**
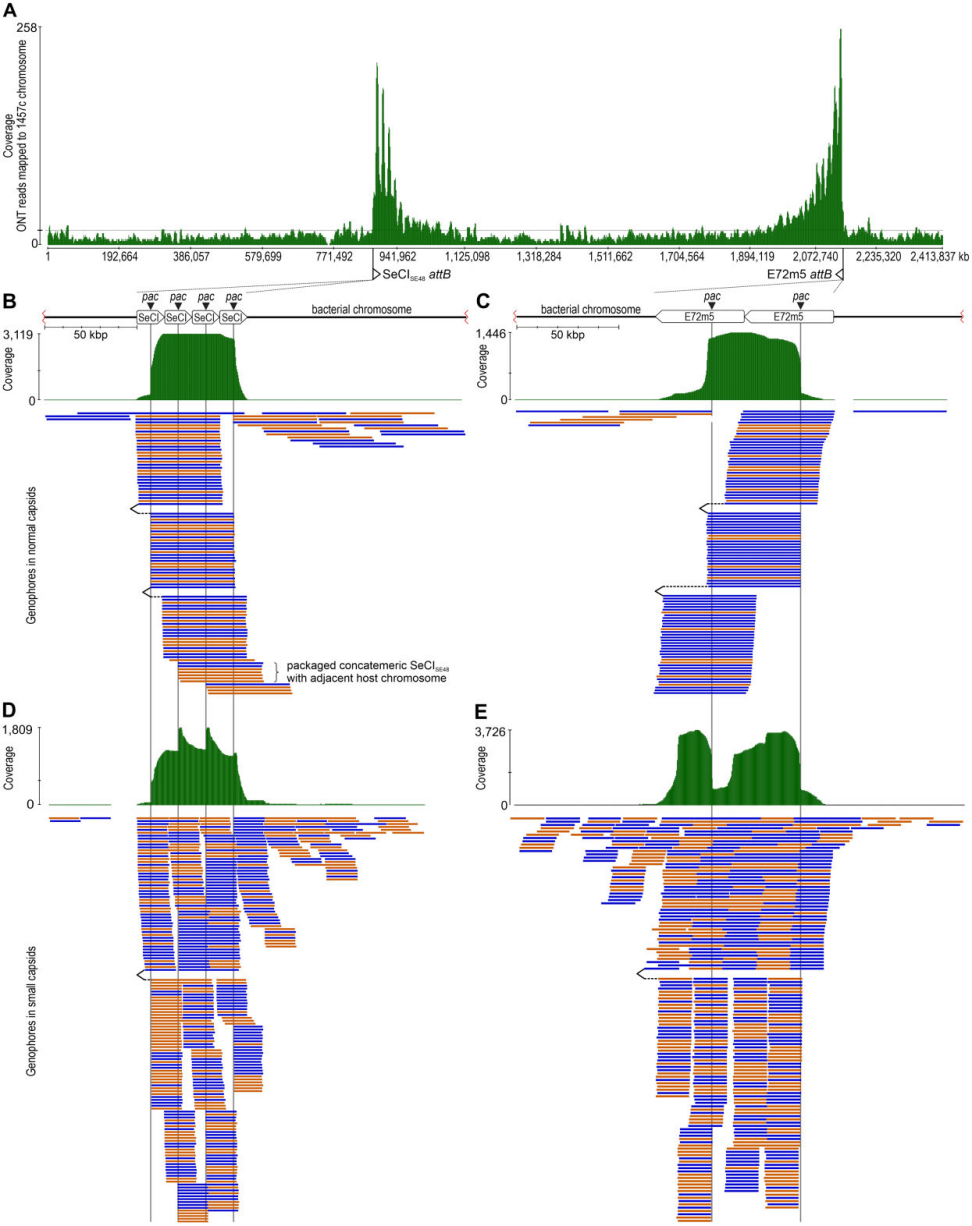
Mapping of sequencing reads to the host, phage, and SeCI_SE48_ genomes. All ONT reads from two biological replicates (NCBI SRA acc. nos. SRR32323971 and SRR32323983) of spontaneous induction in strain 1457LA were mapped to the 1457c genome sequence to eliminate highly covered SeCI_SE48_ and E72m5 regions for clarity (**A**). For both MGEs, the integration site (*attB*) is depicted with an arrow indicating the direction of packaging. The coverage decreases in the direction of the packaging with distance from the MGE position. This corresponds to the frequency of packaging of host sequences into virions due to lateral transduction. To show the sequence organization of SeCI_SE48_ and phage E72m5 complete packaged genophores, these were mapped to multimeric SeCI_SE48_ (**B, D**) or E72m5 (**C, E**) genomes, respectively. A coverage corresponding to the cyclic permutation of the genomes with an inaccurate start of packaging at the *pac* sites can be observed. An integrated multimeric form was used to demonstrate genophores resulting from lateral transduction. Genophores containing both the MGE and host sequence or multiple copies of the MGE and host sequence were present (B–E). This indicates that concatemer formation may also occur prior to MGE excision. This was proved by the occurrence of genophores in normal capsids containing SeCI_SE48_ multimers with an adjacent host chromosome (B). Data obtained after treatment of strain 1457(SeCI_SE48_+) with phage E72m5 (B, C) and ciprofloxacin (D, E) were used for mapping. Only part of the total read count is depicted; the color of the mapped genophores indicates their orientation to the reference (blue for forward; orange for reverse).

Phage and SeCI_SE48_ genophores are terminally redundant and circularly permuted, which results from a headful packaging mechanism. Sequence alignment showed slightly imprecise packaging initiation from the phage and SeCI_SE48_  *pac* sites (Figs 2 and 3). For phage 48, the median size of full-length genophores of normal virions was 43.8 kbp (±2% using 5th–95th percentile range) (Supplementary Table 3B). Relative to the 42.5-kbp unit genome of phage 48, this represents 103.1% (max. 105% using 95th percentile) of its genome. The median size of full-length genophores in phage 48-derived small-headed virions was 15.9 kbp (± 4%), representing 37.4% of the phage unit genome and 107% (max. 111.7%) of the 14.8-kbp SeCI_SE48_ genome (Supplementary Table 3B).

For phage E72m5, the median size of full-length genophores of normal virions was 45.3 kbp (±1%) (Supplementary Table 3B). This corresponds to 102.5% (max. 103.5%) of the 44.2-kbp phage unit genome. The median size of full-length genophores in phage E72m5-derived small-headed virions was 16.5 kbp (± 4%), representing 37.4% of the phage unit genome and 111.5% (max. 115.6%) of the SeCI_SE48_ genome (Supplementary Table 3B). The normal capsid capacity of phages 48 and E72m5 enabled the packaging of a three-copy multimer of the SeCI_SE48_ (Fig. 3).

### Distribution of MGE and host DNA in normal and small-headed particles

We observed packaging of the same concatemers into normal and small proheads for both phages E72m5 and 48 (Fig. 2). This could be explained by the fact that the phage-encoded large terminase subunit is involved in the packaging of both phage and SeCI_SE48_ concatemers. It recognizes and binds to a prohead portal, which is also of phage origin in both cases. Hence the DNA-terminase complex has no specificity to a particular capsid type and can switch between normal and small proheads. Switching between both types of particles during packaging of the same concatemer was observed as an apparent increase in the coverage of sequencing reads from ∼16 kbp downstream of the *pac* site (Fig. 2A), corresponding to the capacity of the small-headed particle (Fig. 2B). The level of observed increase was proportional to the abundance of small particles (Supplementary Table 5).

**Figure 4.**
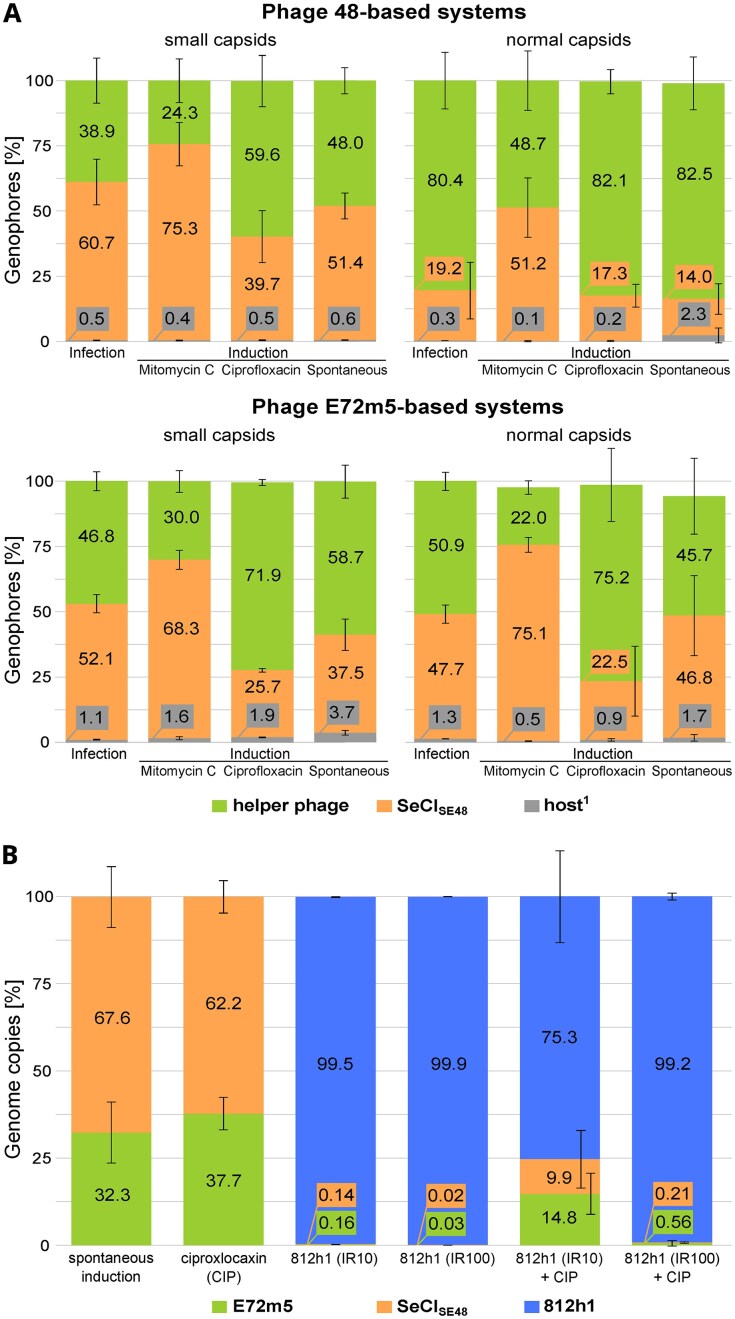
Relative abundance of full-length genophores with different origins in released normal and small-headed particles after helper phage 48 and E72m5 infection and induction (**A**). Relative content of phage and SeCI_SE48_ genome copies in sequencing data obtained from particles released spontaneously and after treatment of strain 1457LA with ciprofloxacin, lytic phage 812h1, and their combination (**B**). The results are presented as means of three biological replicates with standard deviations shown as whiskers. In phage 48-based systems (part A), there was a statistically significant difference in the abundance of SeCI_SE48_ and phage genophores from small particles upon mitomycin C induction, and in genophores from normal particles except for mitomycin C induction. In phage E72m5-based systems, this difference was observed in genophores from small particles except for infection, and from normal particles after mitomycin C and ciprofloxacin induction. Furthermore, a statistically significant difference was observed in the abundance of SeCI_SE48_ and phage genome copies (part B) after spontaneous induction and ciprofloxacin induction. Source data and more detailed results for each replicate are given in Supplementary Tables S4 and S7. ^1^Genophores mapped to chromosomal or plasmid reference.

The relative amount of phage, SeCI_SE48_, and host DNA-derived genophores in each capsid type is given in Supplementary Table S4 for all the analyzed systems. Despite the variability among biological replicates, evident trends can be observed in the distribution of genophores of different origins depending on the helper phage and the agent used for induction (Fig. 4A). After infection or the spontaneous and ciprofloxacin-mediated induction of phage 48, the ratio of phage to SeCI_SE48_ genophores in the released small-headed particles was approximately equimolar (there was no statistically significant difference). In normal-sized particles, phage genophores clearly prevailed. Mitomycin C-mediated induction resulted in a predominance of SeCI_SE48_ genophores in small capsids and equimolar distribution in normal capsids.

After infection, the ratio of phage E72m5 and SeCI_SE48_ genophores was approximately equimolar in both normal and small-headed particles (Fig. 4A). The results were similar for spontaneous induction, where a small but statistically significant difference (*P* < .05) in distribution was observed in the case of small capsids. Ciprofloxacin-mediated induction resulted in an abundance of phage E72m5 genophores in all released virions. In both strains, 1457LA and SE48L, induction with mitomycin C led to a considerable increase in the relative amount of SeCI_SE48_ genophores in small and normal capsids when compared to all other systems.

In both lysogenic strains, the direction of packaging of the helper prophage and SeCI_SE48_ is toward each other, which is reflected in the increased frequency of packaging of chromosomal sequences between these elements due to lateral transduction (Fig. 3A). Rare full-length reads from small-headed particles containing slightly more than one copy of the 15-kb plasmid p1457 (CP020462) of the strains 1457 and 1457LA were detected (Supplementary Table S4). From all systems, only one multimeric read of p1457, with a size of 43.7 kb (slightly below the capacity of the normal particle determined using CBS), was detected in sequencing reads obtained after E72m5 infection (biological replicate SRR32323981). Both cases suggest packaging from a concatemer during plasmid replication. No plasmid sequences from strains SE48 and SE48L were detected as multimers among the full-length reads from small-headed and normal particles. Despite this, using all sequencing reads from each system, the coverage (Minimap2 v2.24 plug-in, Data Type: Oxford Nanopore, K-Mer Length: 28) of an 8-kb pSE48_1 ranged from 1× in infection to 266× in ciprofloxacin induction, which was 7.1 × 10^4^ times and 1.4 × 10^2^ times lower compared to SeCI_SE48_, respectively. The coverage of 1-kb pSE48_2 ranged from 15× in infection to 2206× in ciprofloxacin induction, which was 4.7 × 10^3^ times and 17.3 times lower compared to SeCI_SE48_, respectively.

The relative abundance of normal particles and small-headed particles differed according to the helper phage and its life cycle (Supplementary Table S5). For phage 48, normal particles were slightly overrepresented after infection. For infection with phage E72m5, small-headed particles were almost twice as abundant as normal ones. For both lysogens SE48L and 1457LA, antimicrobial-mediated prophage induction led to a substantial increase in the number of small-headed particles.

### Impact of antimicrobial agents on the induction of MGEs

**Figure 5.**
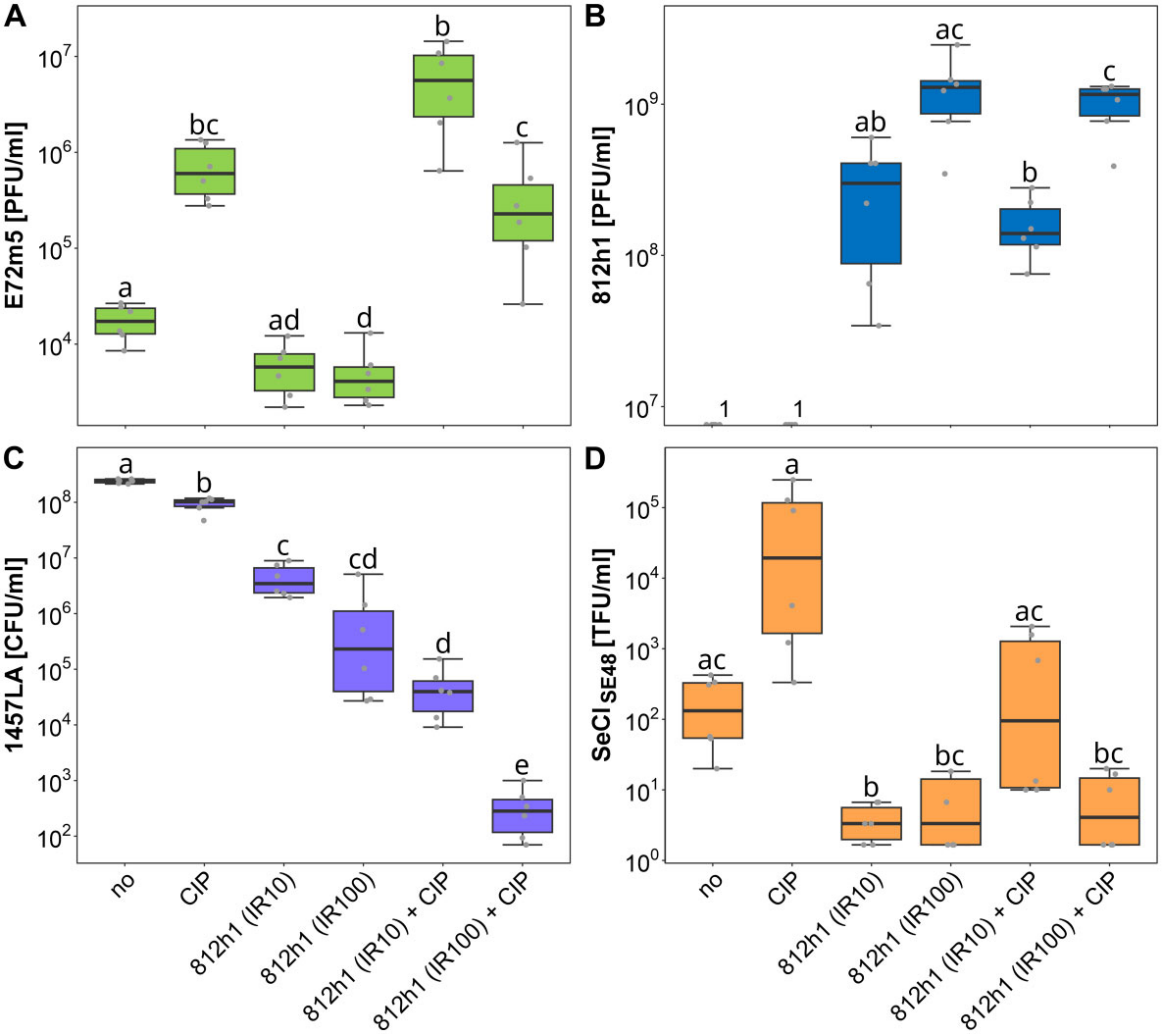
Effect of stress factors on prophage induction, therapeutic phage propagation, bacterial cell count reduction, and SeCI_SE48_ transduction. Counts of released induced phage E72m5 virions (**A**), infecting phage 812h1 virions (**B**), viable cell counts of 1457LA culture (**C**), and SeCI_SE48_-carrying transductant-forming units (**D**) after spontaneous induction (no treatment) and treatment with ciprofloxacin (CIP), phage 812h1 at input ratios (IR) 10 and 100, and their combination. The difference between values was assessed statistically for each system: Different small letters above boxplots indicate statistically significant differences between groups (*P* < .05). Groups sharing at least one letter are not significantly different from each other. Source data and more detailed results are given in Supplementary Tables S8 and S9. ^1^No 812h1 virions were released after spontaneous and ciprofloxacin-mediated induction, as the phage 812h1 was not used.

We further investigated the impact of the *in vitro* treatment of strain 1457LA culture with ciprofloxacin (1× MIC), therapeutic lytic phage 812h1, and their combination on the mobilization of genetic elements. Considering the moderately efficient adsorption (Supplementary Table S6) of phage 812h1 on strain 1457LA cells (60% of virions adsorbed in 20 min), we chose two different input ratios (IR = MOI_INPUT_) of the phage, 10 and 100. In addition to the analysis of sequencing reads ≥500 nt from all released virions (Supplementary Table 7), viable cell count and phage titer were determined on selectively permissive strains *S. aureus* 1137 and *S. cohnii* A6C (Supplementary Table 8). The occurrence of MGE in a total number of virions was expressed as the mean percentage of genome copies calculated as the coverage of a given MGE relative to the sum of the coverage of all reference sequences (Fig. 4B). An increase in the induction of prophage E72m5 was observed whenever ciprofloxacin was used (Fig. 5A). After treatment with ciprofloxacin, the released particles of both types together contained almost 40% of phage E72m5 genome copies and ~60% of SeCI_SE48_ copies (not genophores, as SeCI_SE48_ is packaged into phage particles as a trimer, and the phage genome is incomplete in small-headed particles) (Fig. 4B). This ratio was similar to spontaneous induction, but the use of ciprofloxacin resulted in a significantly higher release of E72m5 virions (Fig. 5A). After treatment with phage 812h1 alone, its genome significantly predominated, making up to 99.9% of genome copies (Fig. 4B). Phage 812h1 infection led to a 100-fold lower titer of the induced phage E72m5 compared to ciprofloxacin treatment (Fig. 5A) and the reduction in induced phage titer was also statistically significant for a higher IR of phage 812h1 compared even to untreated spontaneous induction.

The combination with ciprofloxacin had no effect on the resulting titer of phage 812h1 (Fig. 5B). Compared to the phage 812h1-only treatment, an increased titer of induced phage E72m5 (Fig. 5A) and an increased relative amount of packaged MGEs was detected in the combined treatment using phage 812h1 and ciprofloxacin (Fig. 4), especially at the lower input ratio of phage 812h1. Nevertheless, the number of released phage E72m5 virions in the combined treatment was not statistically significantly different from the ciprofloxacin-only treatment (Fig. 5A). Importantly, the combined treatment led to the highest decrease in the number of viable bacterial cells (Fig. 5C). The application of phage 812h1 at an input ratio of 100 together with ciprofloxacin was statistically significantly the most effective treatment, and resulted in a 6 order of magnitude decrease in viable cell count compared to ciprofloxacin-only or no treatment.

Furthermore, the type of interaction between phage 812h1 and ciprofloxacin was evaluated as synergistic in the inhibition of strain 1457LA (Supplementary Fig. 3A) using SynergyFinder 3.0 [60]. We calculated the overall synergy score as the mean of score values for both the phage IRs used: Bliss and ZIP models 0.35 (*P*-value 4.56 × 10^−9^), HSA model 1.05 (*P*-value 6.68 × 10^−19^), and Loewe additivity model 0.64 (*P*-value 2.76 × 10^−5^). Calculated positive values indicate synergy, while negative values indicate antagonism. The effect of ciprofloxacin on the stability of prophage E72m5 when the culture was co-treated with phage 812h1 was found to be antagonistic (Supplementary Fig. 3B). The scores for each of the models applied were: Bliss -26.67 (*P*-value 1.00 × 10^−5^), ZIP -5.33 (*P*-value 2.54 × 10^−1^), HSA -26.63 (*P*-value 1.03 × 10^−5^), and Loewe -26.63 (*P*-value 1.03 × 10^−5^).

Using strain 1457LA_ermC containing SeCI_SE48_ with an inserted erythromycin resistance gene, we experimentally determined the number of transducing particles released after each treatment (Fig. 5D). We used the *S. cohnii* strain A6C, which is susceptible to the transducing phage E72m5 and resistant to the lytic phage 812h1, as a recipient. About 75% of E72m5 virions adsorbed onto the strain A6C cells, while over 99% adsorbed onto *S. epidermidis* strain 1457 (Supplementary Table S6) within 30 minutes of cocultivation. The relatively low efficiency of adsorption and potential anti-MGE mechanisms may account for the lower number of transductants obtained compared to what was expected based on the abundance of SeCI_SE48_-carrying small-headed and normal particles (Supplementary Tables S4 and S5). The results (Fig. 5D; Supplementary Table S9) showed that the lowest absolute and relative amounts of transducing particles were produced when the lytic phage was used alone or at a higher input ratio in combination with ciprofloxacin. When the antibiotic was combined with a lower input ratio of phage 812h1, the values were comparable to those obtained from spontaneous induction. The highest absolute and relative amounts of transducing particles were detected when ciprofloxacin was used alone.

## Discussion

Despite the fact that several recent sequencing-based studies have focused on transductomics [34, 62, 63], the approaches used have not been able to effectively compare and quantify individual genophores in phage and phage-derived small-headed particles. Previous approaches in staphylococci based on indirect evidence using Southern blot analysis only confirmed the presence of phage and PICI DNA in normal as well as small-headed capsids [32]. Here we present an ONT-based approach for transduced mobilome analysis suitable for the identification of phage, PICI, chromosome, and plasmid genophores in individual particle types after infection with helper phage, induction by SOS-response inducing agents, and/or infection with a lytic phage.

Sequence analysis clearly shows that both phage and SeCI_SE48_ genophores are terminally redundant and circularly permuted, and are therefore packaged via a headful mechanism starting with an imprecise cleavage at the *pac* site. The redundancy of phage genophores in normal particles was consistent with the previously published value of 2%–10% of genome length [64]. The redundancy of SeCI_SE48_ genophores in small-headed particles was greater, 7%–16% of the genome length. Since the capacity of the small heads was 36%–37% of the normal ones for both helper phages, the size plasticity of SeCI_SE48_ genophores enables the packaging of complete PICI into small-headed particles derived from phages with different capsid sizes. At the same time, it probably enables the packaging of various PICIs using the same capsid morphogenesis mechanism.

We confirmed that, besides normal virions, the phage genome is also packaged into small-headed particles, where it is incomplete [32]. The uptake of these incomplete phage DNAs might be an important mechanism of prophage evolution through recombination, as the recipient cell does not undergo phage-mediated lysis. Next, we confirmed the assumption that, besides the small-headed particles, PICIs are packaged into normal virions as trimers [65], and are thus transferred completely by both capsid types.

In normal capsids, multi-copy SeCI_SE48_ genophores including adjacent host sequences formed during lateral transduction were detected (Fig. 3B). This confirms the previously proposed formation of concatemeric islands within the host chromosome when PICI does not undergo the typical chronological order of excision, replication, and packaging of DNA, but the packaging is initiated from the *pac* site before the excision [34]. Detected reads consisting of phage and extensive adjacent chromosomal sequences indicate lateral transduction mediated by delayed prophage excision (Fig. 3C and E). Lateral transduction enables the transfer of chromosomal DNA with a more than 1000 times higher frequency than can be observed in generalized or specialized transduction [15]. Accordingly, the distribution of sequencing reads mapped to the host chromosome (Fig. 3A), and at the same time, the very low number of detected plasmids transmitted by generalized transduction showed that lateral transduction occurred much more frequently than other transduction types.

As shown in lateral transduction, the frequency of packaging decreases with distance from the *pac* site (Fig. 3A). Given the same (headful) mechanism of encapsidation, this probably also applies to phage and PICI concatemers. However, in terms of the frequency of individual gene transfer, the distance of a given gene from the *pac* site is limiting for lateral transduction of the host chromosome, but not for the packaging of concatemers, because the complete set of genes of a given unit genome is packaged in every single packaging event. According to a study on *pac*-phage P22 with a headful packaging mechanism [66, 67], a series of 2–12 packaging events occurs after cleavage at the *pac* site, while the DNA packaging series length increases with time after infection. It is not known whether the number of packaging events in a single series is limited by concatemer length, or whether multiple series can be initiated on a single concatemer molecule [68].

The model system described is generalizable to staphylococcal PICIs mobilized by the *pac*-type helper phages, which contribute most to the formation of transducing particles [69], though some PICIs can utilize *cos*-type helper phages. In this case, PICI carries a *cos* site recognized by the phage TerS, leading to competition between PICI and phage concatemers for the packaging complexes [70]. However, *cos*-type packaging is assumed to mediate specialized transduction only [69]. Recently, capsid-forming PICIs (cf-PICIs) have been reported to be possibly present in staphylococci [71]. Unlike other PICIs, cf-PICIs are completely independent of the phage packaging complex and do not interfere with phage reproduction. Disregarding transduction events, only phage DNA is packaged into phage capsids, while only cf-PICI DNA is packaged into cf-PICI capsids.

The low packaging frequency of the plasmids of strains SE48 and 1457 is consistent with our previous unsuccessful attempts to transduce them [13]. Considering the increased abundance of SE48 plasmid sequences in released virions after ciprofloxacin induction and the fact that no multimeric SE48 plasmids were detected in free virions as a capsid-fulfilling genophore, it should be considered that they could be carried as observed monomers and plasmid fragments with an unknown preference for particle type. The size of small plasmid sequences is insufficient for the headful mechanism of packaging required for the virion assembly [72]. Therefore, we hypothesize that the promiscuity of the packaging machinery may play a role, similarly as in the phage T4, in which DNA is transferred into the finished phage heads in the same way as into the proheads [72], while the presence of a concatemer may not be necessary [73]. Packaging could be facilitated by the linearization of plasmids due to DNA damage by induction agents, which would correspond to the observed notably higher abundance of strain SE48 plasmid sequences in the data from inductions than from infections. Thus, these small DNAs could be co-transduced together with phage or PICI genomes, or fill the capsids in multiple separate copies, as described for phages P1 [74] and T4 [75].

The distribution of phage and island genophores varied depending on the helper phage, its life cycle, and the host strain used. For helper phage 48 and SeCI_SE48_, similar ratios were observed in a given particle type across systems, except for induction by mitomycin C. The fact that phage 48 genophores clearly predominated in normal particles while island abundance increased in small-headed particles indicates a co-evolutionary trade-off that allows the efficient propagation of both elements. This is consistent with the fact that phage 48 has been repeatedly propagated on strain SE48, naturally containing the island SeCI_SE48_, since the 70’s [39]. In contrast, the ratios of phage E72m5 and SeCI_SE48_ genophores in normal and small particles within each system were similar. The difference in genophore distribution after infection and induction may be caused by an observed deletion in prophage E72m5 at position 42 828–43 309 bp of the reference (GenBank acc. no. PV036961). This resulted in the fusion gene consisting of parts of *orf70* and *orf72*. The function of the corresponding genes is not known, but they are located in the region between *rinB* and *rinA* genes, which affect DNA packaging into procapsids [76].

During DNA packaging, two types of terminase complexes are formed, consisting of phage-encoded large subunit (TerL) and small subunit (TerS) encoded either by the phage or by SeCI_SE48_. Since the specificity of the terminase complex is determined by the TerS, and phage and SeCI_SE48_ encode distinct TerS proteins that recognize their own *pac* sites, there is probably no competition between these two types of terminase complexes for DNA concatemers. However, SeCI_SE48_ encodes the Ppi (phage auxiliary encapsidation inhibitor) protein [13], which can reduce the efficiency of phage DNA packaging by binding to phage TerS [31]. It remains unclear how this affects the phages used. Both phages encode identical small terminase subunits, however, the sequences starting with a putative *pac* site upstream of the corresponding gene differ (71.8% identity). The different efficiency of phage and SeCI_SE48_ genome recognition by the terminase complexes can explain the differences in the genophore distribution in both helper phages. Small-headed particles formed a much larger proportion of the virions released after any E72m5 induction or infection than in phage 48. Short co-evolution may have played a role in easier SeCI_SE48_ induction in strain 1457LA, leading to a higher expression of island-encoded genes compared to SE48L.

Mitomycin C and ciprofloxacin efficiently increased titers of induced phages when compared to spontaneous induction, but had different impacts on the ratio of packaged genophores. Mitomycin C-mediated induction significantly increased the portion of SeCI_SE48_ genophores, forming a majority in both types of particles for both helper prophages. While mitomycin C acts via cross-linking strands of the DNA double helix [77], ciprofloxacin inhibits DNA replication by blocking DNA gyrase and topoisomerase IV [78]. The resulting double-stranded DNA breaks cause an SOS response [79] and thus an excision of the helper prophage. Triggering its lytic cycle then leads to the mobilization of PICI [80]. The *S. aureus* pathogenicity island (SaPI) operon I containing the *terS* gene encoding a small terminase subunit can be induced by the SOS response of the host cell, and thus, the packaging of bacterial DNA from pseudo-*pac* sites can appear even if the phage is not the helper phage of the SaPI and the SaPI is not mobilized [65]. Based on this, we suppose that the reason for the effect of mitomycin C could be the additional expression of the SeCI_SE48_ operon, which encodes TerS and capsid modulation proteins, which could be expressed directly via the SOS response even before PICI derepression and excision. The different induction outcomes may be related to the higher MIC multiple used for mitomycin C compared to ciprofloxacin or to a different mechanism of action. The effect of mitomycin C and ciprofloxacin on PICI mobilization has already been studied in *S. aureus*, with the conclusion that both agents enhance island transfer [80]. However, these conclusions were based on the presence of genophores of small-headed particles without further differentiation of their origin. Therefore, they rather support our conclusion that both agents can increase the relative abundance of small-headed particles.

The treatment of the staphylococcal culture with ciprofloxacin led to increased production of infectious particles of induced prophage, as described previously [17]. Despite the reported upregulation of prophage gene expression upon the phage K infection of *S. aureus* [81], treatment with the closely related therapeutic phage 812h1 led to a drastic reduction in the titer of the released temperate phage compared to ciprofloxacin use. The upregulation of prophage gene expression can be explained by the SOS response caused by the host DNA damage by phage 812h1 nucleases, which causes prophage excision. However, intracellular temperate phage DNA awaiting packaging may subsequently be damaged by phage 812h1 host-takeover mechanisms, resulting in low numbers of released mature and infectious temperate phage virions (Fig. 5A). Therefore, purely complete genophore reads could not be used for analysis due to a shortage of them caused by the fragmentation and high abundance of phage 812h1 sequencing data (Fig. 4B).

Although the combined treatment led to an increase in both the titer of induced phage and the relative amount of packaged mobilome compared to treatment with phage alone, the values observed were not statistically significantly higher than those observed for the treatment with ciprofloxacin alone. Most probably, this is because with a lower input ratio of therapeutic phage, a lower number of cells are infected in the initial phase, allowing the induction of prophage and subsequently PICI in the remaining uninfected bacterial population due to the SOS response triggered by ciprofloxacin.

Importantly, the combined treatment led to a six-order-of-magnitude decrease in the number of viable bacterial cells, and it was more effective than the antibiotic or therapeutic phage alone. Based on the results obtained using various interaction models, we proved that this interaction was synergistic. Similarly, ciprofloxacin with phages has already been used to eradicate *S. aureus* or *Pseudomonas aeruginosa* based on phage-antibiotic synergy [82, 83]. Therefore, this combined approach appears to be the best in terms of treatment efficacy while not increasing the risk of spreading resistance and virulence genes compared to standard antibiotic treatment. The application of lytic phage has been shown to reduce the risk of MGE transduction to levels comparable to spontaneous induction, or even significantly lower, depending on the quantity of phage used and its combination with antibiotics. Nevertheless, the advantages of therapy combining lytic phage and ciprofloxacin need to be confirmed *in vivo*.

This work contributes to the elucidation of the interactions between phages, PICIs, and host DNA packaging; it significantly advances our understanding of the biology of transduction and provides a robust framework for assessing the risks associated with horizontal gene transfer in therapeutic contexts.

## Supplementary Material

gkaf1165_Supplemental_File

## Data Availability

The data underlying this article are available in the article, its online supplementary materials, and in subject-specific public repositories. Sequencing data are deposited within BioProject PRJNA1198866 (https://www.ncbi.nlm.nih.gov/bioproject/PRJNA1198866), except for the genome of phage E72m5 deposited under GenBank acc. no. PV036961. Whole-genome sequences of *S. epidermidis* strains obtained in this work are deposited under the following GenBank acc. nos.: SE48L (CP180175), 1457(SeCI_SE48_+) (CP180176), 1457LA (CP180177), 1457LA_ermC (CP194848), and 1457c (CP180178). BioSample accession numbers and Sequence Read Archive (SRA) data for biological replicates are listed in Supplementary Tables S3 and S7. The following sequences taken from the GenBank database were also used in this paper: *S. epidermidis* strains SE48 (CP066303) and 1457 (CP020463); SeCI_SE48_ (MW368309); phage 48 (MW364972) and prophage 1457 (CP020463); plasmids pSE48_1 (CP066304), pSE48_2 (CP066305), and p1457 (CP020462). The results of testing the robustness of the CBS method under different parameters are accessible on Zenodo (https://doi.org/10.5281/zenodo.17054370).
