# Supplementary material for: Virion content unpacked by long-read sequencing: stress-induced changes in transmitted staphylococcal mobilome due to phage-satellite interactions
Source: Nucleic Acids Res. 2025 Nov 8;53(20):gkaf1165. doi: 10.1093/nar/gkaf1165 (PMC12597102; doi:10.1093/nar/gkaf1165)
Supplement: gkaf1165_Supplemental_File [file gkaf1165_supplemental_file.pdf]

Supporting Information – Supplementary Figures & Tables

Virion content unpacked by long-read sequencing: Stress-induced changes in transmitted staphylococcal mobilome due to phage-satellite interactions

Tibor Botka[a], Soňa Smetanová[a,b], Adam Vinco[a], Eliška Kučerová[a], Kristína Rovňáková[a], Alena Siváková[c], Ivana Mašlaňová[a] and Roman Pantůček[a]

[a]Department of Experimental Biology, Faculty of Science, Masaryk University, Brno, 625 00, Czech Republic

[b]RECETOX, Masaryk University, 625 00, Brno, Czech Republic

[c]Department of Microbiology, St Anne's University Hospital and Faculty of Medicine, Masaryk University, Brno, 625 00, Czech Republic

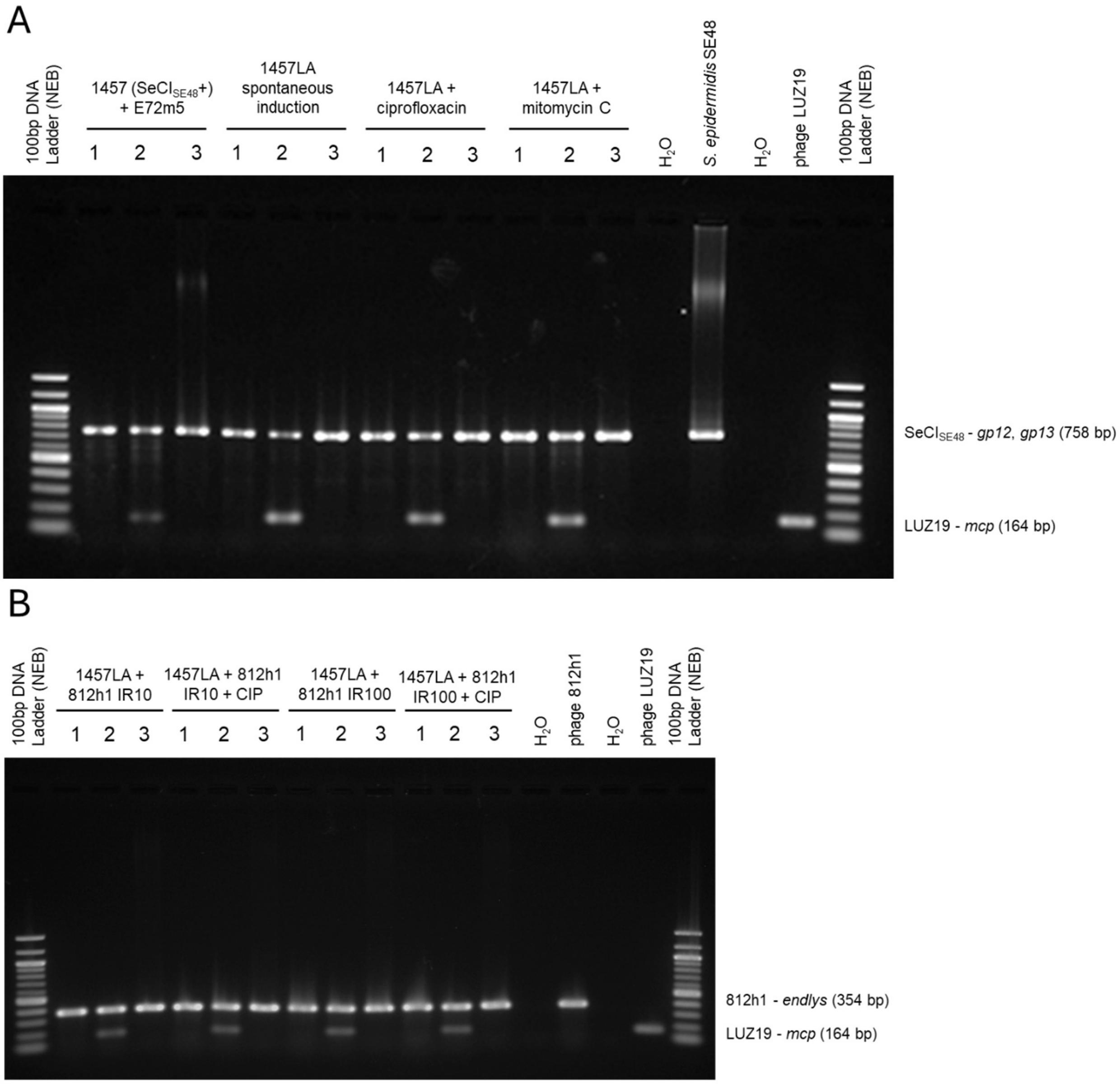

1 – virion suspension before external DNA addition and DNase treatment
2 – virion suspension after addition of phage LUZ19 DNA
3 – isolated virion DNA (incl. DNase treatment)

Supplementary Figure 1: Confirmation of extra-virion DNA removal using external DNA of phage LUZ19. PCR targeting SeCI$_{SE48}$ (A) and lytic phage 812h1 (B) served as internal controls. All biological replicates were tested using this method.

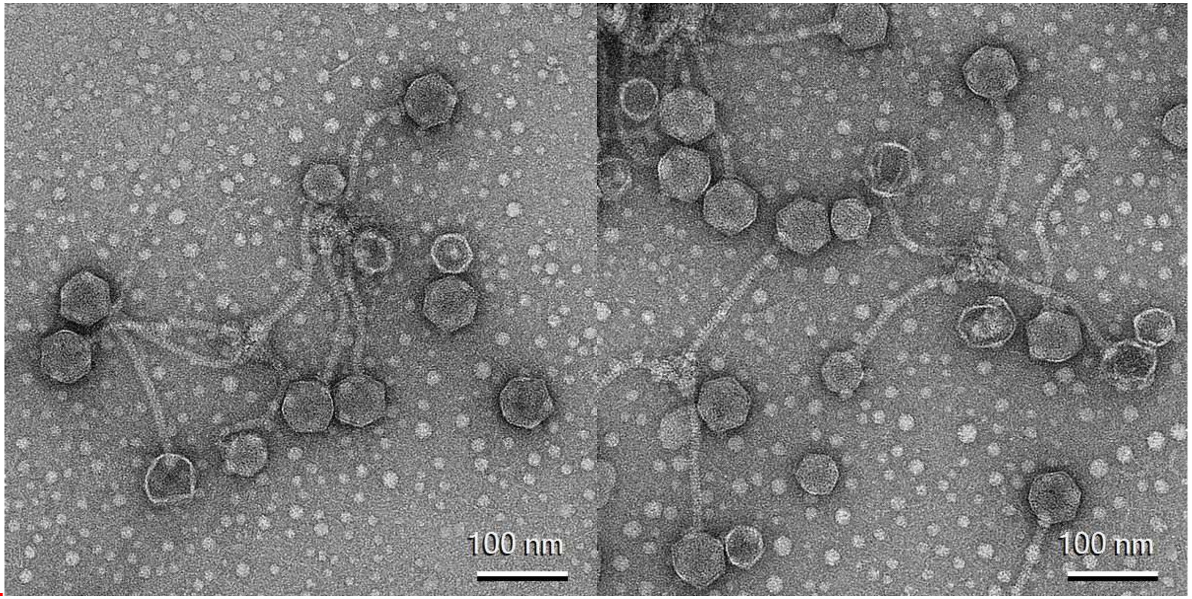

Supplementary Figure 2: Transmission electron microscope image of negatively stained normal and small-headed virions of phage E72m5 propagated on *S. epidermidis* 1457($SeCI_{SE48}$+).

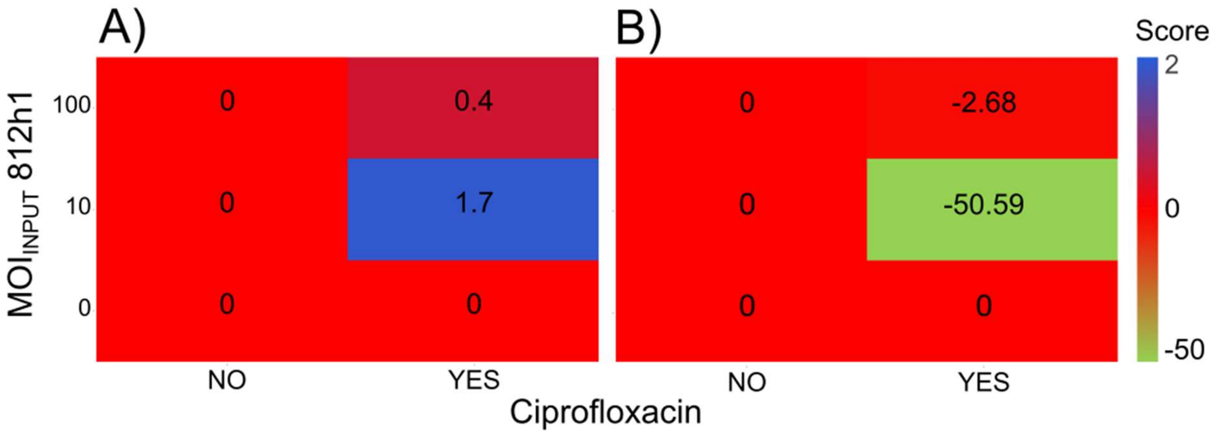

Supplementary Figure 3: Evaluation of interaction between phage 812h1 and ciprofloxacin in inhibition of strain 1457LA (A) and stability of prophage E72m5 (B) using the Highest single agent (HSA) model. Positive values indicate synergy and negative values indicate antagonism.

Supplementary Table 1: Primers used for *S. epidermidis* genome editing.

Name	Sequence	Purpose	Target sequence (GenBank acc. no.)	Target position (bp)	Reference
phi1457_del_F	tattGTCGACcctattgtgtttgttccagtt	Amplification of empty *att*-site of prophage phi1457 with adjacent regions	*S. epidermidis* strain 1457LA (CP180177.1)	1807759-1809106	this study
phi1457_del_R	tctaCCCGGGacctattttacttacgcctctt				this study
ErmC_1_F	GCGTTGAAATAGGAATTGAATGAGACATGCTACAC	Amplification of erythromycin resistance cassette	Staphylococcal shuttle vector pCN51 (KR781468.1)	2101-3361	this study
ErmC_1_R	TCTTTATTATTAAGAAAACTGGTTTAAGCCGAC				this study
ErmC_LHA_1_F	CTGGGTACCGGGCCCCCCCTCGAGGTCGACTAACAGAGAAGCAAGGGTC	Amplification of left homology arm	*S. epidermidis* strain 1457LA (CP180177.1)	889053-889867	this study
ErmC_LHA_1_R	TCTCATTCAATTCCTATTTCAACGCAACACAAAAAC				this study
ErmC_RHA_1_F	AAACCAGTTTTCTTAATAATAAAGACATAATGGAACTATTTTTAG	Amplification of right homology arm	*S. epidermidis* strain 1457LA (CP180177.1)	891129-891938	this study
ErmC_RHA_1_R	GGCCGCTCTAGAACTAGTGGATCCCCCGGGCAGTATTTTCATCCGCATACAC				this study

Supplementary Table 2: Titers (PFU/ml) of phages induced by selected antibiotics.

Conc.	ATB	Source strain / induced phage / titration strain									
		1457LA / phage E72m5 / 1457					SE48L / phage 48 / SE48				
		mean	technical replicates			SD	mean	technical replicates			SD
0×MIC	GEN	4.10E+03	4.40E+03	3.90E+03	3.90E+03	2.40E+02	1.30E+04	1.30E+04	1.30E+04	1.20E+04	4.70E+02
	OXA	3.90E+03	3.20E+03	3.80E+03	4.60E+03	5.70E+02	1.40E+04	1.20E+04	1.30E+04	1.60E+04	1.70E+03
	CIP	5.50E+03	5.40E+03	5.50E+03	5.60E+03	8.20E+01	1.80E+04	1.60E+04	1.60E+04	2.20E+04	2.80E+03
0.5×MIC	GEN	3.20E+03	3.30E+03	2.80E+03	3.60E+03	3.30E+02	4.60E+03	3.60E+03	4.80E+03	5.40E+03	7.50E+02
	OXA	1.90E+03	1.70E+03	1.80E+03	2.10E+03	1.70E+02	5.70E+03	4.00E+03	6.00E+03	7.00E+03	1.20E+03
	CIP	4.00E+05	3.60E+05	3.80E+05	4.50E+05	3.90E+04	5.30E+05	3.00E+05	6.00E+05	7.00E+05	1.70E+05
0.75×MIC	GEN	3.30E+03	2.40E+03	3.70E+03	3.90E+03	6.60E+02	2.60E+03	2.80E+03	2.80E+03	2.30E+03	2.40E+02
	OXA	2.50E+03	1.90E+03	2.40E+03	3.20E+03	5.40E+02	1.10E+04	6.00E+03	1.00E+04	1.60E+04	4.10E+03
	CIP	1.10E+06	5.00E+05	1.30E+06	1.60E+06	4.60E+05	1.80E+06	1.00E+06	2.00E+06	2.50E+06	6.20E+05
1×MIC	GEN	2.60E+03	2.00E+03	2.70E+03	3.20E+03	4.90E+02	2.90E+03	2.90E+03	3.10E+03	2.70E+03	1.60E+02
	OXA	2.40E+03	2.00E+03	2.60E+03	2.70E+03	3.10E+02	9.70E+03	7.00E+03	1.00E+04	1.20E+04	2.10E+03
	CIP	1.00E+06	9.00E+05	1.00E+06	1.10E+06	8.20E+04	2.10E+06	1.50E+06	1.80E+06	3.00E+06	6.50E+05
2mg/l	MITC	1.90E+06	2.00E+06	1.90E+06	1.70E+06	1.00E+05	2.00E+07	8.10E+06	3.90E+07	1.30E+07	1.40E+07

MIC – minimum inhibitory concentration. GEN – gentamycin (inhibition of proteosynthesis and membrane damage); OXA - oxacillin (inhibition of cell wall); CIP – ciprofloxacin and MITC – mitomycin C (damage of DNA). For 1457LA, MICs were GEN: 0.5 mg/l; OXA: 0.125 mg/l; CIP: 0.25 mg/l; MITC: 0.25 mg/l. For SE48L, MICs were GEN: 0.5 mg/l; OXA: 0.25 mg/l; CIP: 0.5 mg/l; MITC: 0.5 mg/l.

Supplementary Table 3: The assessment of sequencing reads of all systems (A) and the determination of genophore size ranges (B).

A)

Sequencing set	BioSample (NCBI)	Biological replicate (SRA)	Total no. of reads	Genophores in small-headed capsids						Genophores in normal capsids				
				Background sequences [%][1]	Length range by CBS [bp]		Median	Max. median difference[2]	Max. Cliff's Delta [absolute value][3]	Length range by CBS [bp]		Median	Max. median difference[2]	Max. Cliff's Delta [absolute value][3]
					min	max				min	max			
Infection of SE48 by phage 48	SAMN45855537	SRR32323966	158807	0.91	15018	16809	15860	16 (<0.1%)	0.02	42860	44771	43822	165 (0.4%)	0.13
		SRR32323978	78227	1.17	15078	16585	15875			42560	45132	43839		
		SRR33893563	92207	0.30	15054	16925	15860			42523	44799	43674		
SE48L treatment by mitomycin C	SAMN45855539	SRR32323964	72575	0.31	15041	16544	15702	63 (0.4 %)	0.11	42776	44145	43491	100 (0.2%)	0.13
		SRR32323976	48747	1.21	15101	16460	15758			42497	44952	43584		
		SRR34537063	57701	0.56	15022	16578	15765			42658	44468	43591		
SE48L treatment by ciprofloxacin	SAMN45855538	SRR32323965	135717	2.22	15035	16282	15695	170 (1.1 %)	0.3	42449	44238	43334	423 (1%)	0.52
		SRR32323977	114951	4.30	15101	16664	15825			43193	44360	43757		
		SRR35012806	430912	0.51	15244	16469	15865			N/D	N/D	N/D		
Spontaneous induction in SE48L	SAMN45867298	SRR32323970	17610	0.51	15154	16178	15674	117 (0.7 %)	0.27	N/D	N/D	N/D	29 (<0.1%)	0.07
		SRR34942316	50374	1.19	15209	16531	15790			42939	44208	43566		
		SRR34942363	25773	0.58	15233	16454	15791			43129	43919	43537		
Infection of 1457c by E72m5	SAMN45867299	SRR31745133	109877	N/A - strain 1457c does not carry $SeCI_{SE48}$						44743	45791	45265	6 (<0.1%)†	0.004†
Infection of 1457(SeCI_SE48+) by E72m5	SAMN45855534	SRR32323969	131973	0.44	15562	17520	16499	52 (0.3%)	0.08	44869	45884	45355	131 (0.3%)	0.19
		SRR32323981	141380	0.34	15656	17633	16536			44732	45796	45286		
		SRR33894003	116688	0.83	15611	17715	16484			44325	46033	45224		
1457LA treatment by mitomycin C	SAMN45855536	SRR32323967	86049	1.07	15713	16738	16226	39 (0.2%)	0.09	N/D	N/D	N/D	N/A	N/A
		SRR32323979	33472	1.71	15729	16744	16243			N/D	N/D	N/D		
		SRR34538127	174857	3.38	15695	16815	16265			N/D	N/D	N/D		
1457LA treatment by ciprofloxacin	SAMN45855535	SRR32323968	57855	1.48	15899	17020	16379	58 (0.4%)	0.1	N/D	N/D	N/D	N/A	N/A
		SRR32323980	77117	1.60	15723	17210	16435			44334	45804	45108		
		SRR34539423	290182	2.85	15820	17317	16437			N/D	N/D	N/D		
Spontaneous induction in 1457LA	SAMN45867297	SRR32323971	50346	1.44	15660	16703	16208	1016 (6.2%)	0.86	N/D	N/D	N/D	N/A	N/A
		SRR32323983	122270	4.62	14047	16287	15192			N/D	N/D	N/D		
		SRR35012763	42012	3.00	15677	16620	16158			N/D	N/D	N/D		

[1]Exponential regression model based on sequencing data of each biological replicate with length >=1000 and <=40000 (without genophores from small-headed particles) was used to predict the number of background sequences with ambiguous origins.

[2]Maximum absolute difference in median of biological replicates and relative difference as a percentage of the overall median for a given phage/SeCI (**Supplementary Table 3B**).

[3]Evaluation of the difference between the sequencing read lengths in biological replicates according to Cliff's Delta method: negligible <0.15; small <0.33; medium <0.47; large ≥0.47.

†Values comparing data from E72m5 phage infection of strain 1457c to data of the whole system of infection of strain 1457(SeCI_SE48+).

N/D - not determinable due to low sequencing yields or high fragmentation of the sample; N/A - not applicable.

B)

Phage	Length median in bp (5th - 95th perc)	
	Small capsid genophore	Normal capsid genophore
48	15859 (15290 - 16537)	43786 (42878 - 44602)
E72m5	16502 (15932 - 17116)	45269 (44789 - 45735)[1]

The length of the genophores was determined based on data from all biological replicates of each infection system.

[1]Determined using data from infections of phage E72m5 on strains 1457(SeCI_SE48+) and 1457c.

Supplementary Table 4: Relative amount of genophores with different origins packaged in normal and small-headed particles.

System (type / agens / strain)	Biological replicate (SRA)	small capsid genophores										normal capsid genophores									
		total no.	phage		SeCI		host (plasmid)[1]		unmapped[2]		total no.	phage		SeCI		host (plasmid)[1]		unmapped[2]			
			no.	%	no.	%	no.	%	no.	%		no.	%	no.	%	no.	%	no.	%		
infection / phage 48 / SE48	SRR32323966	17 620	6 293	35.72%	11 234	63.76%	90	0.51%	3	0.02%	8 386	6 794	81.02%	1 562	18.63%	30	0.36%	0	0.00%		
	SRR32323978	4 689	2 380	50.76%	2 293	48.90%	15	0.32%	1	0.02%	13 632	12 734	93.41%	852	6.25%	46	0.34%	0	0.00%		
	SRR33893563	23 372	7 038	30.11%	16 204	69.33%	128	0.55%	2	0.01%	9 007	6 023	66.87%	2 955	32.81%	29	0.32%	0	0.00%		
induction / mitomycin C / SE48L	SRR32323964	23 966	3 089	12.89%	20 798	86.78%	76	0.32%	3	0.01%	801	261	32.58%	539	67.29%	0	0.00%	1	0.12%		
	SRR32323976	7 382	2 409	32.63%	4 945	66.99%	16	0.22%	12	0.16%	618	353	57.12%	265	42.88%	0	0.00%	0	0.00%		
	SRR34537063	16 363	4 469	27.31%	11 786	72.03%	103	0.63%	5	0.03%	1 591	896	56.32%	689	43.31%	6	0.38%	0	0.00%		
induction / ciprofloxacin / SE48L	SRR32323965	7 299	3 467	47.50%	3 787	51.88%	36	0.49%	9	0.12%	915	760	83.06%	150	16.39%	4	0.44%	1	0.11%		
	SRR32323977	10 956	6 538	59.68%	4 359	39.79%	28	0.26%	31	0.28%	1 118	974	87.12%	140	12.52%	3	0.27%	1	0.09%		
	SRR35012806†	1 162	832	71.60%	319	27.45%	8	0.69%	3	0.26%	312	237	75.96%	72	23.08%	0	0.00%	3	0.96%		
spontaneous induction / SE48L	SRR32323970†	2 880	1 185	41.15%	1 672	58.06%	21	0.73%	2	0.07%	189	129	68.25%	42	22.22%	12	6.35%	6	3.17%		
	SRR34942316	6 215	3 104	49.94%	3 076	49.49%	31	0.50%	4	0.06%	832	741	89.06%	88	10.58%	3	0.36%	0	0.00%		
	SRR34942363	4 366	2 313	52.98%	2 031	46.52%	20	0.46%	2	0.05%	650	587	90.31%	60	9.23%	2	0.31%	1	0.15%		
infection / phage E72m5 / 1457c	SRR31745133	strain 1457c does not carry $SeCI_{SE48}$									1 903	1 899	99.79%	0	0.00%	4	0.21%	0	0.00%		
infection / phage E72m5 / 1457($SeCI_{SE48}$)	SRR32323969	42 947	22 141	51.55%	20 410	47.52%	374 (2)	0.87%	22	0.05%	2 278	1 086	47.67%	1 162	51.01%	29	1.27%	1	0.04%		
	SRR32323981	41 824	19 282	46.10%	22 083	52.80%	446 (2)	1.07%	13	0.03%	4 269	2 108	49.38%	2 102	49.24%	59	1.38%	0	0.00%		
	SRR33894003	31 476	13 484	42.84%	17 606	55.93%	379	1.20%	7	0.02%	4 715	2 624	55.65%	2 026	42.97%	64	1.36%	1	0.02%		
induction / mitomycin C / 1457LA	SRR32323967†	7 519	2 044	27.18%	5 329	70.87%	142 (1)	1.89%	4	0.05%	478	89	18.62%	375	78.45%	3	0.63%	11	2.30%		
	SRR32323979†	2 290	821	35.85%	1 447	63.19%	19	0.83%	3	0.13%	634	158	24.92%	453	71.45%	3	0.47%	20	3.15%		
	SRR34538127†	5 215	1 403	26.90%	3 697	70.89%	107 (1)	2.05%	8	0.15%	237	53	22.36%	179	75.53%	1	0.42%	4	1.69%		
induction / ciprofloxacin / 1457LA	SRR32323968†	3 817	2 695	70.61%	1 002	26.25%	72	1.89%	48	1.26%	388	312	80.41%	63	16.24%	6	1.55%	7	1.80%		
	SRR32323980	13 594	9 984	73.44%	3 359	24.71%	232	1.71%	19	0.14%	656	584	89.02%	67	10.21%	5	0.76%	0	0.00%		
	SRR34539423†	15 581	11 174	71.72%	4 058	26.04%	332	2.13%	17	0.11%	775	434	56.00%	318	41.03%	4	0.52%	19	2.45%		
spontaneous induction / 1457LA	SRR32323971†	3 414	1 933	56.62%	1 377	40.33%	103	3.02%	1	0.03%	148	91	61.49%	49	33.11%	3	2.03%	5	3.38%		
	SRR32323983†	5 013	3 370	67.23%	1 464	29.20%	157	3.13%	22	0.44%	69	34	49.28%	27	39.13%	0	0.00%	8	11.59%		
	SRR35012763†	1 021	532	52.11%	439	43.00%	50	4.90%	0	0.00%	129	34	26.36%	88	68.22%	4	3.10%	3	2.33%		

[1]The total number of reads mapped to host sequences (chromosome and plasmid), of which the number of reads mapped to the plasmid(s) is shown in parentheses.

[2]Not mapped due to multiple targets (caused by lateral transduction) or sequencing errors.

†As the CBS could not be used for the size range determination of complete genophores from normal capsids (see **Supplementary Table 3**), reads of 25 - 46 kbp were analyzed (thus, no two or more reads could be part of the same genophore, and excessively long reads resulting from sequencing errors or chimeras were excluded).

The full-length genophore size ranges were determined using CBS (**Supplementary Table 3**). Genophores were mapped to references using Geneious mapper; Minimum quality mapping: 10 (90% confidence); Maximum Gap Size: 500 bp, Maximum Gaps Per Read: 20%, Word length: 10, Max. Mismatches Per Read: 20%, Fine Tuning: None.

References used for phage 48-based systems: phage 48 (MW364972), $SeCI_{SE48}$ (MW368309), SE48 (CP066303) with deleted sequence of $SeCI_{SE48}$, and plasmids pSE48_1 (CP066304) and pSE48_2 (CP066305).

References used for phage E72m5-based systems: phage E72m5 (PV036961), prophage E72m5 (CP180177; pos. 2,161,688 - 2,205,368 bp), $SeCI_{SE48}$ (MW368309), 1457c (CP180178) and plasmid p1457 (CP020462).

Supplementary Table 5: Analysis of DNA samples using Agilent Fragment Analyzer.

System	Biological replicate	Small-headed particles					Normal particles			
		CPA	molar correction	amount %	mean	SD	CPA	amount %	mean	SD
infection / phage 48 / SE48	1	22.792	62.952	42	46.58	4.38	87.200	58	53.42	4.38
	2	50.279	138.871	45			167.269	55		
	3	1389.686	3838.313	52			3480.402	48		
induction / mitomycin C / SE48L	1	12.596	34.790	76	70.64	4.25	11.165	24	29.36	4.25
	2	15.37	42.452	71			17.413	29		
	3	1408.196	3889.437	65			2067.096	35		
induction / ciprofloxacin / SE48L	1	14.687	40.565	68	66.71	1.71	18.725	32	33.29	1.71
	2	9.882	27.294	65			14.696	35		
	3	N/D	N/A	N/A			N/D	N/A		
spontaneous induction / SE48L	1	12.266	33.879	82	74.23	5.51	7.482	18	25.77	5.51
	2	0.97	2.679	69			1.190	31		
	3	1.707	4.715	72			1.876	28		
infection / phage E72m5 / 1457(SeCI$_{SE48+}$)	1	72.654	198.999	63	62.49	1.11	117.778	37	37.51	1.11
	2	15.322	41.967	64			23.958	36		
	3	857.26	2348.035	61			1501.467	39		
induction / mitomycin C / 1457LA	1	67.347	184.463	86	87.72	3.94	30.820	14	12.28	3.94
	2	20.972	57.442	84			10.736	16		
	3	606.374	1660.858	93			120.686	7		
induction / ciprofloxacin / 1457LA	1	174.141	476.972	94	88.14	5.27	29.578	6	11.86	5.27
	2	23.177	63.482	81			14.581	19		
	3	448.233	1227.710	89			152.787	11		
spontaneous induction / 1457LA	1	1.156	3.166	51	44.37	5.26	3.087	49	55.63	5.26
	2	1.909	5.229	45			6.464	55		
	3	6.735	18.447	38			30.396	62		

CPA - corrected peak area obtained using the software PROSize 2.0.0.51 (Advanced Analytical Technologies) with automatic initial peak delimitation and subsequent manual correction.

Molarity correction of values of small-headed particles: CPA * normal genophore length / small-headed genophore length.

N/D - not determinable; N/A - not applicable.

Supplementary Table 6: Adsorption assays.

A) Adsorption assay of phage 812h1 on *S. epidermidis* strain 1457LA in MPB.

Time [min]	Phage 812h1 titer on *S. aureus* strain 1137 [PFU/ml]					Non-adsorbed phage 812h1 virions [%]				
	mean	technical replicates			SD	mean	technical replicates			SD
0	1.42E+09	1.53E+09	1.44E+09	1.29E+09	9.90E+07	100.00	107.75	101.41	90.85	6.97
2	1.25E+09	1.21E+09	1.32E+09	1.22E+09	4.97E+07	88.03	85.21	92.96	85.92	3.5
5	1.19E+09	1.05E+09	1.32E+09	1.19E+09	1.10E+08	83.57	73.94	92.96	83.8	7.77
10	8.87E+08	9.50E+08	9.40E+08	7.70E+08	8.26E+07	62.44	66.9	66.2	54.23	5.81
15	6.53E+08	6.00E+08	8.30E+08	5.30E+08	1.28E+08	46.01	42.25	58.45	37.32	9.03
20	5.80E+08	5.50E+08	6.30E+08	5.60E+08	3.56E+07	40.85	38.73	44.37	39.44	2.51

B) Adsorption assay of phage E72m5 on *S. epidermidis* strain 1457 in BHI.

Time [min]	Phage E72m5 titer on *S. epidermidis* strain 1457 [PFU/ml]					Non-adsorbed phage E72m5 virions [%]				
	mean	technical replicates			SD	mean	technical replicates			SD
0	2.41E+08	2.57E+08	2.24E+08	2.42E+08	1.35E+07	100.00	106.64	92.95	100.41	5.60
2	7.97E+07	8.00E+07	8.20E+07	7.70E+07	2.05E+06	33.06	33.20	34.02	31.95	0.85
5	2.27E+07	2.23E+07	2.28E+07	2.30E+07	2.94E+05	9.42	9.25	9.46	9.54	0.12
10	7.87E+06	7.50E+06	7.60E+06	8.50E+06	4.50E+05	3.26	3.11	3.15	3.53	0.19
20	2.12E+06	2.03E+06	2.32E+06	2.02E+06	1.39E+05	0.88	0.84	0.96	0.84	0.06
30	9.60E+05	8.40E+05	1.09E+06	9.50E+05	1.02E+05	0.40	0.35	0.45	0.39	0.04

C) Adsorption assay of phage E72m5 on *S. cohnii* strain A6C in BHI.

Time [min]	Phage E72m5 titer on *S. epidermidis* strain 1457 [PFU/ml]					Non-adsorbed phage E72m5 virions [%]				
	mean	technical replicates			SD	mean	technical replicates			SD
0	2.41E+08	2.57E+08	2.24E+08	2.42E+08	1.35E+07	100.00	106.64	92.95	100.41	5.60
2	2.15E+08	2.06E+08	2.18E+08	2.20E+08	6.18E+06	89.07	85.48	90.46	91.29	2.57
5	1.79E+08	1.60E+08	2.06E+08	1.72E+08	1.95E+07	74.41	66.39	85.48	71.37	8.08
10	1.55E+08	1.62E+08	1.43E+08	1.60E+08	8.52E+06	64.32	67.22	59.34	66.39	3.54
20	1.01E+08	9.50E+07	9.50E+07	1.12E+08	8.01E+06	41.77	39.42	39.42	46.47	3.33
30	6.07E+07	4.90E+07	6.50E+07	6.80E+07	8.34E+06	25.17	20.33	26.97	28.22	3.46

Supplementary Table 7: Origin of DNA in phage particles after treatment with different agents on strain 1457LA.

Induction agens (strain 1457LA)	BioSample (NCBI)	Biological replicate (SRA)	Reads ≥ 500nt total no.	Reads ≥ 500nt total no. of nt	Mapped nt no.	Mapped nt % of total[1]	812h1 (150 582 bp) no. of nt	812h1 % nt[2]	812h1 % ref.[3]	812h1 mean cov.	812h1 % genome copies[4]
no	SAMN45867297	SRR32323971	50 343	360 750 823	360 705 374	99.99	0	0	0	0	0
		SRR32323983	112 556	625 769 335	623 870 872	99.70	0	0	0	0	0
		SRR35012763	38 266	229 333 075	229 203 425	99.94	0	0	0	0	0
ciprofloxacin (CIP)	SAMN45855535	SRR32323968	57 537	374 010 407	372 135 722	99.50	0	0	0	0	0
		SRR32323980	76 771	797 520 910	797 351 745	99.98	0	0	0	0	0
		SRR34539423	266 551	1 717 195 887	1 716 960 201	99.99	0	0	0	0	0
812h1 (IR10)	SAMN45855540	SRR32323963	39 083	215 527 340	214 832 196	99.68	214 607 882	99.89559	100	1 425.18948	99.58173
		SRR32323975	25 040	210 957 617	210 476 332	99.77	210 111 918	99.82686	100	1 395.33223	99.43627
		SRR33745109	370 839	819 127 479	817 441 750	99.79	816 395 616	99.87202	100	5 421.60163	99.51036
812h1 (IR10) + CIP	SAMN45855542	SRR32323961	50 624	281 815 016	279 957 492	99.34	251 394 941	89.79754	100	1 669.48866	63.24905
		SRR32323973	32 241	260 884 517	260 304 467	99.78	239 675 781	92.07517	100	1 591.66289	73.39810
		SRR33745053	392 046	868 699 719	867 044 613	99.81	842 068 415	97.11939	100	5 592.09212	89.32324
812h1 (IR100)	SAMN45855541	SRR32323962	39 877	510 972 309	510 239 828	99.86	510 166 583	99.98564	100	3 387.96525	99.94257
		SRR32323974	60 758	505 536 158	503 938 193	99.68	503 812 925	99.97514	100	3 345.77124	99.91084
		SRR33744784	316 870	746 332 870	744 880 402	99.81	744 673 188	99.97218	100	4 945.30016	99.92643
812h1 (IR100) + CIP	SAMN45855543	SRR32323960	27 584	291 086 455	290 608 788	99.84	290 564 169	99.98465	100	1 929.60758	99.90309
		SRR32323972	50 247	407 285 766	405 260 223	99.50	403 235 319	99.50034	100	2 677.84542	98.08472
		SRR33743890	206 297	595 610 113	594 430 782	99.80	593 985 522	99.92509	100	3 944.59844	99.66578

Biological replicate (SRA)	E72m5 (44 155 bp) no. of nt	E72m5 % nt[2]	E72m5 % ref.[3]	E72m5 mean cov.	E72m5 % genome copies[4]	SeCI_SE48 (14 816 bp) no. of nt	SeCI_SE48 % nt[2]	SeCI_SE48 % ref.[3]	SeCI_SE48 mean cov.	SeCI_SE48 % genome copies[4]
SRR32323971	198 968 355	55.16091	100	4 506.13419	31.13862	147 341 466	40.84815	100	9 944.75337	68.72097
SRR32323983	417 786 589	66.96684	100	9 461.81834	43.52694	181 462 792	29.08659	100	12 247.75864	56.34302
SRR35012763	100 941 018	44.03993	100	2 286.06088	22.22167	118 325 813	51.62480	100	7 986.35347	77.63139
SRR32323968	233 267 247	62.68338	100	5 282.91806	38.38158	125 487 059	33.72078	100	8 469.69891	61.53426
SRR32323980	531 382 509	66.64342	100	12 034.48101	42.09303	245 083 157	30.73714	100	16 541.78975	57.85826
SRR34539423	985 224 400	57.38190	100	22 312.86151	32.84664	675 282 440	39.33012	100	45 577.91847	67.09499
SRR32323963	93 122	0.04335	84.6	2.10898	0.14736	45 155	0.02102	98.5	3.04772	0.21295
SRR32323975	123 233	0.05855	100	2.79092	0.19889	12 558	0.00597	73.4	0.84760	0.06040
SRR33745109	344 003	0.04208	100	7.79081	0.14300	110 516	0.01352	100	7.45923	0.13691
SRR32323961	20 815 082	7.43509	100	471.40940	17.85948	7 381 761	2.63674	100	498.22901	18.87555
SRR32323973	17 688 601	6.79535	100	400.60245	18.47342	2 588 684	0.99448	100	174.72219	8.05716
SRR33745053	22 099 351	2.54881	100	500.49487	7.99447	2 462 173	0.28397	100	166.18338	2.65447
SRR32323962	39 514	0.00774	49.8	0.89489	0.02640	11 266	0.00221	76.2	0.76039	0.02243
SRR32323974	62 506	0.01240	85	1.41560	0.04227	14 687	0.00291	91.7	0.99129	0.02960
SRR33744784	72 951	0.00979	81.6	1.65216	0.03338	16 582	0.00223	73.3	1.11920	0.02261
SRR32323960	23 351	0.00804	35.7	0.52884	0.02738	19 890	0.00684	71.9	1.34247	0.06950
SRR32323972	1 803 623	0.44505	100	40.84754	1.49617	162 284	0.04004	100	10.95329	0.40120
SRR33743890	270 888	0.04557	99.2	6.13493	0.15501	93 662	0.01576	100	6.32168	0.15973

Biological replicate (SRA)	p1457 (15 142 bp) no. of nt	p1457 % nt[2]	p1457 % ref.[3]	p1457 mean cov.	p1457 % genome copies[4]	1457c (2 413 837 bp) no. of nt	1457c % nt[2]	1457c % ref.[3]	1457c mean cov.	1457c % genome copies[4]
SRR32323971	218 739	0.06064	100	14.44585	0.09982	14 176 814	3.93030	93.5	5.87314	0.04059
SRR32323983	275 315	0.04413	100	18.18221	0.08364	24 346 176	3.90244	95.7	10.08609	0.04640
SRR35012763	167 614	0.07313	100	11.06948	0.10760	9 768 980	4.26214	80.9	4.04708	0.03934
SRR32323968	93 428	0.02511	100	6.17012	0.04483	13 287 988	3.57074	63.3	5.41284	0.03933
SRR32323980	82 543	0.01035	100	5.45126	0.01907	20 803 536	2.60908	83.1	8.47428	0.02964
SRR34539423	247 801	0.01443	100	16.36514	0.02409	56 205 560	3.27355	96.2	23.28474	0.03428
SRR32323963	12 104	0.00563	53.2	0.79937	0.05585	73 933	0.03441	2.7	0.03012	0.00210
SRR32323975	63 668	0.03025	100	4.20473	0.29964	164 955	0.07837	6.1	0.06719	0.00479
SRR33745109	170 382	0.02084	100	11.25228	0.20653	421 233	0.05153	13.6	0.17451	0.00320
SRR32323961	4 132	0.00148	27.4	0.27288	0.01034	361 576	0.12915	9.1	0.14729	0.00558
SRR32323973	21 383	0.00821	63.9	1.41216	0.06512	330 018	0.12678	9.6	0.13443	0.00620
SRR33745053	23 922	0.00276	88.8	1.57984	0.02524	390 752	0.04507	11.1	0.16188	0.00259
SRR32323962	4 305	0.00084	28.5	0.28431	0.00839	18 160	0.00356	0.6	0.00740	0.00022
SRR32323974	8 522	0.00169	44.1	0.56281	0.01681	39 553	0.00785	1.6	0.01611	0.00048
SRR33744784	12 507	0.00168	41.5	0.82598	0.01669	105 174	0.01412	4.1	0.04357	0.00088
SRR32323960	0	0	0	0	0	1 378	0.00047	0.1	0.00056	0.00003
SRR32323972	7 084	0.00175	36.6	0.46784	0.01714	51 913	0.01281	2.1	0.02115	0.00077
SRR33743890	11 240	0.00189	67	0.74231	0.01876	69 470	0.01169	3.2	0.02878	0.00073

[1]Unmapped nucleotides originated from reads with multiple hits (reads resulting from lateral transduction or chimeric reads resulting from a ligation step during library preparation) or due to mismatches, e.g., sequencing errors.

[2]Percentage of mapped nucleotides was calculated as the number of mapped nucleotides for a given reference divided by the sum of mapped nucleotides for all references.

[3]Completeness of reference sequence coverage.

[4]Amount of sequencing data of a given genome relative to the genome size and expressed as a percentage of genome copies (not genophores) was calculated as coverage of a given reference divided by the sum of coverage of all reference genomes.

Only reads with a size above 500nt and q-score > 10 were mapped. Settings were as follows: Mapper = Geneious; Minimum quality mapping: 10 (90% confidence); Maximum Gap Size: 200 bp, Maximum Gaps Per Read: 100%, Word length: 6, Max. Mismatches Per Read: 50%, Fine Tuning: None.

Supplementary Table 8: Titer (PFU/ml) of phages E72m5 and 48, and viable cell count (CFU/ml) of *S. epidermidis* strain 1457LA after induction by different treatments (A), and the statistical evaluation (B).

A)

Agens	PFU/ml (on titration strain[1])												CFU/ml			
	E72m5 + 812h1 (1457)				E72m5 (A6C)				812h1 (1137)				1457LA			
	biol. replicates	median	5th percentile	95th percentile	biol. replicates	median	5th percentile	95th percentile	biol. replicates	median	5th percentile	95th percentile	biol. replicates	median	5th percentile	95th percentile
	1.83E+04				2.66E+04				0.00E+00				2.62E+08			
	5.27E+04				2.18E+04				0.00E+00				2.40E+08			
no (spontaneous induction)	3.14E+04	1.81E+04	1.22E+04	4.74E+04	2.43E+04	1.78E+04	9.52E+03	2.60E+04	0.00E+00	0.00E+00	0.00E+00	0.00E+00	2.43E+08	2.42E+08	2.16E+08	2.63E+08
	1.17E+04				8.53E+03				0.00E+00				2.20E+08			
	1.38E+04				1.37E+04				0.00E+00				2.14E+08			
	1.78E+04				1.25E+04				0.00E+00				2.63E+08			
	1.00E+06				1.35E+06				0.00E+00				1.12E+08			
	1.89E+06				1.26E+06				0.00E+00				7.97E+07			
ciprofloxacin (CIP)	5.40E+05	6.70E+05	5.60E+05	1.67E+06	7.13E+05	6.10E+05	2.90E+05	1.33E+06	0.00E+00	0.00E+00	0.00E+00	0.00E+00	4.70E+07	1.02E+08	5.52E+07	1.17E+08
	7.03E+05				2.77E+05				0.00E+00				1.03E+08			
	6.20E+05				5.07E+05				0.00E+00				1.18E+08			
	6.37E+05				3.30E+05				0.00E+00				1.01E+08			
	1.10E+06				2.20E+03				6.03E+08				2.53E+06			
	2.18E+06				2.90E+03				2.21E+08				7.40E+06			
phage 812h1 (IR=10)	6.83E+05	1.18E+06	5.96E+05	2.15E+06	1.22E+04	5.90E+03	2.38E+03	1.12E+04	6.50E+07	3.14E+08	4.20E+07	5.54E+08	2.30E+06	3.63E+06	2.03E+06	8.55E+06
	2.07E+06				4.67E+03				4.07E+08				4.73E+06			
	1.25E+06				8.17E+03				3.43E+07				1.94E+06			
	5.67E+05				7.13E+03				4.07E+08				8.93E+06			
	3.03E+06				6.07E+03				1.23E+09				1.43E+06			
	4.87E+06				4.93E+03				7.70E+08				2.90E+04			
phage 812h1 (IR=100)	1.41E+06	3.73E+06	1.55E+06	5.47E+06	1.31E+04	4.17E+03	2.38E+03	1.13E+04	3.47E+08	1.30E+09	4.53E+08	2.22E+09	2.70E+04	3.09E+05	2.75E+04	4.18E+06
	5.67E+06				2.60E+03				1.36E+09				5.10E+06			
	1.97E+06				3.40E+03				1.45E+09				1.04E+05			
	4.43E+06				2.30E+03				2.47E+09				5.13E+05			
	1.57E+06				6.40E+05				2.80E+08				9.10E+03			
	1.58E+07				8.53E+06				2.24E+08				1.53E+05			
phage 812h1 (IR=10) + CIP	1.22E+07	8.29E+06	1.95E+06	1.49E+07	1.44E+07	6.12E+06	9.85E+05	1.35E+07	7.53E+07	1.40E+08	8.50E+07	2.66E+08	4.13E+04	3.97E+04	1.02E+04	1.32E+05
	5.47E+06				3.70E+06				1.30E+08				3.80E+04			
	3.07E+06				2.02E+06				1.50E+08				7.00E+04			
	1.11E+07				1.09E+07				1.14E+08				1.35E+04			
	2.20E+06				2.61E+04				1.31E+09				3.43E+02			
	7.33E+06				1.26E+06				7.73E+08				7.00E+01			
phage 812h1 (IR=100) + CIP	8.37E+05	2.89E+06	1.18E+06	6.83E+06	5.40E+05	2.33E+05	4.53E+04	1.08E+06	3.90E+08	1.17E+09	4.86E+08	1.30E+09	9.33E+01	2.88E+02	7.58E+01	8.75E+02
	5.33E+06				2.77E+05				1.07E+09				1.00E+03			
	2.60E+06				1.88E+05				1.26E+09				2.33E+02			
	3.17E+06				1.03E+05				1.26E+09				5.00E+02			

[1]*S. aureus* strain 1137 is sensitive only to phage 812h1 and resistant to phage E72m5; *S. cohnii* strain A6C is sensitive to phage E72m5 and resistant to phage 812h1.

EOP of phage E72m5 on *S. cohnii* strain A6C (vs *S. epidermidis* 1457) was 0.67.

EOP of phage 812h1 on *S. epidermidis* strain 1457 (vs *S. aureus* 1137) was 0.002.

EOP of phage 812h1 on *S. epidermidis* strain 1457LA (vs *S. aureus* 1137) was 0.15

The outlying values that were not used in the testing are marked in red.

B)

E72m5 (A6C) (PFU/ml)

GAMES-HOWELL POST-HOC TEST

group1	group2	estimate	p.adj	p.adj.signif
CIP	IR10	-4.767881	2.11E-06	****
CIP	IR100	-4.9307206	1.46E-06	****
CIP	IR100CIP	-1.0633783	5.50E-01	ns
CIP	IR10CIP	1.940571	6.30E-02	ns
CIP	no	-3.621303	1.96E-05	****
IR10	IR100	-0.1628396	9.97E-01	ns
IR10	IR100CIP	3.7045028	4.00E-03	**
IR10	IR10CIP	6.708452	2.37E-05	****
IR10	no	1.146578	5.10E-02	ns
IR100	IR100CIP	3.8673423	3.00E-03	**
IR100	IR10CIP	6.8712916	2.07E-05	****
IR100	no	1.3094176	2.40E-02	*
IR100CIP	IR10CIP	3.0039492	2.00E-02	*
IR100CIP	no	-2.5579248	3.10E-02	*
IR10CIP	no	-5.561874	2.07E-04	***

ANOVA TEST

	degrees of freedom	F	p	
Agens	5	6.02E+01	9.86E-15	***
Residuals	30			

812h1 (1137) (PFU/ml)

GAMES-HOWELL POST-HOC TEST

group1	group2	estimate	p.adj	p.adj.signif
IR10	IR100	1.73162968	0.051	ns
IR10	IR100CIP	1.76170206	0.045	*
IR10	IR10CIP	-0.25749249	0.955	ns
IR100	IR100CIP	0.03007238	1	ns
IR100	IR10CIP	-1.98912217	0.001	***
IR100CIP	IR10CIP	-2.01919455	0.000117	***

ANOVA TEST

	degrees of freedom	F	p	
Agens	3	1.27E+01	8.73E-05	***
Residuals	19			

1457LA (CFU/ml)

GAMES-HOWELL POST-HOC TEST

group1	group2	estimate	p.adj	p.adj.signif
CIP	IR10	-3.2620209	2.46E-04	***
CIP	IR100	-5.972845	7.00E-03	**
CIP	IR100CIP	-12.9063965	5.11E-06	****
CIP	IR10CIP	-7.9564915	3.65E-05	****
CIP	no	0.8555066	2.15E-04	***
IR10	IR100	-2.7108241	1.54E-01	ns
IR10	IR100CIP	-9.6443756	2.02E-07	****
IR10	IR10CIP	-4.6944706	9.86E-05	****
IR10	no	4.1175274	1.11E-04	***
IR100	IR100CIP	-6.9335515	1.00E-03	***
IR100	IR10CIP	-1.9836465	4.05E-01	ns
IR100	no	6.8283515	4.00E-03	**
IR100CIP	IR10CIP	4.949905	8.52E-05	****
IR100CIP	no	13.761903	5.38E-06	****
IR10CIP	no	8.811998	2.96E-05	****

ANOVA TEST

	degrees of freedom	F	p	
Agens	5	1.27E+02	<2e-16	***
Residuals	29			

ANOVA with Games-Howell post-hoc test was used for statistical evaluation (logarithms of the values were used).

p: p-value

p.adj: p-value adjusted by Benjamini–Hochberg method

p.adj.signif: significance codes: ns = not significant ($p \geq 0.05$)

* = $p < 0.05$

** = $p < 0.01$

*** = $p < 0.001$

**** = $p < 0.0001$

Supplementary Table 9: Titer (PFU/ml) of phage E72m5 in transducing lysates from various treatments, the number of transductant-forming units (TFU/ml) obtained using the *S. cohnii* strain A6C as a recipient, and the frequency of transduction for each biological replicate (A), and the statistical evaluation (B).

A)

Agens	PFU/ml E72m5 (A6C) biol. replicates	PFU/ml median	PFU/ml 5th percentile	PFU/ml 95th percentile	TFU/ml $SeCI_{SE48}$ (A6C) biol. replicates	TFU/ml median	TFU/ml 5th percentile	TFU/ml 95th percentile	Frequency of transduction % (TFU/PFU) biol. replicates	median	5th percentile	95th percentile
**no (spontaneous induction)**	1.83E+04	1.05E+04	2.48E+03	2.04E+04	4.23E+02	1.83E+02	2.83E+01	4.01E+02	2.31	1.93	0.92	2.29
	2.10E+04				3.08E+02				1.47			
	1.89E+04				3.35E+02				1.78			
	2.73E+03				2.00E+01				0.73			
	2.40E+03				5.33E+01				2.22			
	2.73E+03				5.67E+01				2.07			
**ciprofloxacin (CIP)**	2.13E+06	1.26E+06	1.59E+05	2.93E+06	1.27E+05	4.76E+04	5.55E+02	2.17E+05	5.97	1.88	0.35	10.45
	3.20E+06				9.10E+04				2.84			
	2.07E+06				2.47E+05				11.94			
	1.63E+05				3.33E+02				0.20			
	4.43E+05				4.10E+03				0.92			
	1.57E+05				1.22E+03				0.78			
**phage 812h1 (IR=10)**	1.30E+03	6.00E+02	1.87E+02	1.43E+03	0.00E+00	3.33E+00	0.00E+00	6.67E+00	0.00	1.17	0.00	3.12
	1.47E+03				0.00E+00				0.00			
	1.00E+03				6.67E+00				0.67			
	1.87E+02				6.67E+00				3.57			
	1.87E+02				3.33E+00				1.79			
	2.00E+02				3.33E+00				1.67			
**phage 812h1 (IR=100)**	1.63E+03	1.08E+03	5.12E+02	2.25E+03	1.83E+01	3.33E+00	0.00E+00	1.83E+01	1.12	0.16	0.00	1.04
	2.30E+03				1.83E+01				0.80			
	2.10E+03				6.67E+00				0.32			
	5.17E+02				0.00E+00				0.00			
	5.10E+02				0.00E+00				0.00			
	5.23E+02				0.00E+00				0.00			
**phage 812h1 (IR=10) + CIP**	3.60E+04	1.33E+04	1.57E+03	4.83E+04	1.57E+03	3.47E+02	1.00E+01	1.95E+03	4.36	1.85	0.45	4.26
	2.40E+04				6.82E+02				2.84			
	5.23E+04				2.07E+03				3.96			
	1.65E+03				1.00E+01				0.61			
	2.50E+03				1.00E+01				0.40			
	1.54E+03				1.33E+01				0.87			
**phage 812h1 (IR=100) + CIP**	3.47E+03	1.52E+03	5.28E+02	4.39E+03	2.00E+01	5.00E+00	0.00E+00	1.92E+01	0.58	0.11	0.00	0.66
	2.43E+03				1.67E+01				0.68			
	4.70E+03				1.00E+01				0.21			
	5.97E+02				0.00E+00				0.00			
	5.10E+02				0.00E+00				0.00			
	5.83E+02				0.00E+00				0.00			

B)

SeCISE48 (TFU/ml)				
**DUNN'S POST-HOC TEST**				
**group1**	**group2**	**mean.rank.diff**	**p.adj**	**p.adj.signif**
CIP	no	7.583333	0.2747	ns
IR10	no	-17.75	0.0105	*
IR100	no	-13.583333	0.0553	.
IR100CIP	no	-12.166667	0.0811	.
IR10CIP	no	-1.583333	0.8114	ns
IR10	CIP	-25.333333	0.0003	***
IR100	CIP	-21.166667	0.0027	**
IR100CIP	CIP	-19.75	0.0044	**
IR10CIP	CIP	-9.166667	0.1838	ns
IR100CIP	IR10CIP	-10.583333	0.1243	ns
IR100CIP	IR100	1.416667	0.8114	ns
IR10CIP	IR100	12	0.0811	.
IR100	IR10	4.166667	0.557	ns
IR100CIP	IR10	5.583333	0.4337	ns
IR10CIP	IR10	16.166667	0.0194	*
**Kruskal-Wallis rank sum test**				
	**degrees of freedom**	**H**	**p**	
	5	2.69E+01	6.08E-05	***

Frequency of transduction (TFU/PFU)				
**Kruskal-Wallis rank sum test**				
	**degrees of freedom**	**H**	**p**	
	5	4.04E+00	5.43E-01	ns

Kruskal-Wallis rank-sum test with Dunn´s post-hoc test was used for statistical evaluation.

p: p-value

p.adj: p-value adjusted by Benjamini–Hochberg method

p.adj.signif: significance codes:
ns = $p \geq 0.1$ (not significant)
. = $p < 0.1$ (not significant)
* = $p < 0.05$
** = $p < 0.01$
*** = $p < 0.001$
